# CHIP-dependent regulation of the actin cytoskeleton is linked to neuronal cell membrane integrity

**DOI:** 10.1016/j.isci.2021.102878

**Published:** 2021-07-17

**Authors:** Catarina Dias, Erisa Nita, Jakub Faktor, Ailish C. Tynan, Lenka Hernychova, Borivoj Vojtesek, Jesper Nylandsted, Ted R. Hupp, Tilo Kunath, Kathryn L. Ball

**Affiliations:** 1 Institute of Genetics and Cancer, University of Edinburgh, Edinburgh EH4 2XU, UK; 2 Centre for Regenerative Medicine, Institute for Stem Cell Research, School of Biological Sciences, The University of Edinburgh, Edinburgh EH16 4UU, UK; 3 Research Centre for Applied Molecular Oncology, Masaryk Memorial Cancer Institute, 656 53 Brno, Czech Republic; 4 Membrane Integrity Group, Danish Cancer Society Research Center, Strandboulevarden 49, 2100, Copenhagen, Denmark; 5 University of Gdansk, International Centre for Cancer Vaccine Science, 80-822 Gdansk, Poland

**Keywords:** cell biology, organizational aspects of cell biology, bioinformatics, omics, proteomics

## Abstract

CHIP is an E3-ubiquitin ligase that contributes to healthy aging and has been characterized as neuroprotective. To elucidate dominant CHIP-dependent changes in protein steady-state levels in a patient-derived human neuronal model, CHIP function was ablated using gene-editing and an unbiased proteomic analysis conducted to compare knock-out and wild-type isogenic induced pluripotent stem cell (iPSC)-derived cortical neurons. Rather than a broad effect on protein homeostasis, loss of CHIP function impacted on a focused cohort of proteins from actin cytoskeleton signaling and membrane integrity networks. In support of the proteomics, CHIP knockout cells had enhanced sensitivity to induced membrane damage. We conclude that the major readout of CHIP function in cortical neurons derived from iPSC of a patient with elevate α-synuclein, Parkinson's disease and dementia, is the modulation of substrates involved in maintaining cellular “health”. Thus, regulation of the actin cytoskeletal and membrane integrity likely contributes to the neuroprotective function(s) of CHIP.

## Highlights

• Cortical neurons were generated from iPSC of a patient with Parkinson's disease with ablated CHIP
• SWATH-MS identified a focused cohort of CHIP modulated proteins
• CHIP loss impacts actin cytoskeleton signaling and membrane integrity networks
• CHIP is protective for induced membrane damage

## Graphical abstract

**Figure:**
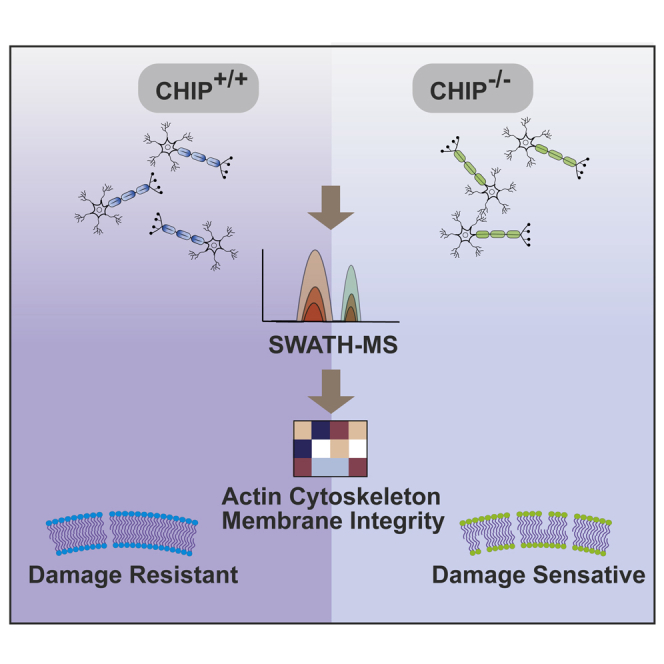

## Introduction

C-terminus of HSC70 Interacting Protein (CHIP) is an E3-ubiquitin ligase originally identified by virtue of its ability to interact with HSC70 (Ballinger et al., 1999). CHIP has historically been studied as part of the molecular chaperone network and has been ascribed a direct role in determining the balance between protein folding, refolding, aggregation, and degradation (Connell et al., 2001; Kampinga et al., 2003; Kevei et al., 2017; Marques et al., 2006; Murata et al., 2001; Zhang et al., 2015). The degree to which CHIP-mediated ubiquitination of unfolded and “damaged” heat shock protein (HSP)-client proteins contributes to the overall physiological function of this E3-ligase remains unclear. CHIP-dependent modulation of the core chaperone machinery is, however, closely related to the ability of CHIP to protect cells and animals from death during thermal stress. When stress is applied, for example, in the form of hyperthermic conditions, loss of CHIP function leads to apoptosis at the cellular level and rapid death in mouse models (Dai et al., 2003). Mechanistically, in response to hyperthermic conditions, heat shock transcription factor (HSF1)-containing chaperone complexes can be activated by CHIP-dependent mechanisms leading to increasing HSP70 levels (Dai et al., 2003). *De Novo* synthesis of HSP70 produces sufficient core chaperone activity to “buffer” the cellular environment and prevent irreversible cellular damage (Dai et al., 2003). It is therefore not surprising that CHIP deletion in mouse model increases susceptibility to proteotoxic stress (Dai et al., 2003; Min et al., 2008). A recent study demonstrated some degree of cell-type variability in the response to loss of CHIP function. Whilst fibroblasts from patients with ataxia (SCAR16), expressing mutant CHIP, had an impaired heat shock response, the response in induced pluripotent stem cell (iPSC)-derived neurons from the same patients was relatively unaffected (Schuster et al., 2020).

Until recently, less attention has been paid to what may be described as the non-canonical function(s) of CHIP (Landré et al., 2013, 2017; Narayan et al., 2011; Tawo et al., 2017). In addition to its HSP-dependent functions, CHIP also has a chaperone-independent activity, the best characterized substrate for which is the interferon regulated transcription factor interferon regulatory factor-1 (IRF-1) (Landré et al., 2017; Narayan et al., 2011). The role of CHIP in IRF-1 regulation, as well as in other key components of the immune system (Kettern et al., 2011; Yang et al., 2011) provides insights into the way this E3-ligase contributes to immune regulation (Zhan et al., 2017). Studies in animal models have identified additional native substrates for CHIP, such as the insulin receptor (INSR) that link directly to organismal health (Adachi et al., 2007; Al-Ramahi et al., 2006; Morishima et al., 2008; Ronnebaum et al., 2013; Tawo et al., 2017; Zhao et al., 2012). Thus, the main activity of CHIP in unstressed cells appears to be closely linked to the maintenance of cellular and organismal “health”, through the specific regulation of native proteins (Al-Ramahi et al., 2006; Alberti et al., 2002; Ronnebaum et al., 2013; Tawo et al., 2017).

In patients with α-synucleinopathies, such as supranuclear palsy, multiple system atrophy, dementia with Lewy bodies, and Parkinsons disease (Mark, 2001), CHIP is downregulated by sequestration into large α-synuclein aggregates or Lewy bodies (Shin et al., 2005). However, less is known about the role of CHIP in earlier disease stages, where Lewy bodies are not detected and CHIP remains soluble. To obtain an α-synucleinopathy model, relevant to early stage disease, we used an established patient-derived iPSC line (AST previously established by (Devine et al., 2011) as a starting point. This line carries a triplication of the α-synuclein-encoding gene, *SNCA*, which results in four copies of *SNCA* and a doubling of the mRNA and protein expression levels in the absence of large synuclein aggregates or Lewy body structures (Devine et al., 2011). Increased steady state levels of α-synuclein are a common background across familial and sporadic α-synucleinopathies (Lashuel et al., 2013). CHIP function was ablated in AST23 iPSC using gene-editing to generate isogenic cells. Following differentiation of the iPSC into cortical neurons — the cell type affected in Lewy body dementia (Gibb et al., 1989) — an unbiased investigation was undertaken to define dominant steady state proteomic changes mediated by CHIP loss, using label-free Sequential Window Acquisition of all Theoretical Mass Spectra (SWATH-MS) (Huang et al., 2015; Rosenberger et al., 2014). Target discovery was centered around two main criteria: disease-relevance and the identification of proteomic changes at early disease stages (in the absence of proteotoxic stress). Systems analysis highlights a role for CHIP in maintaining the actin cytoskeleton, as well as providing a link between CHIP, the cytoskeleton, and membrane integrity.

## Results

### Deletion of CHIP expression in patient-derived iPSC and generation of cortical neurons

**Figure 1:**
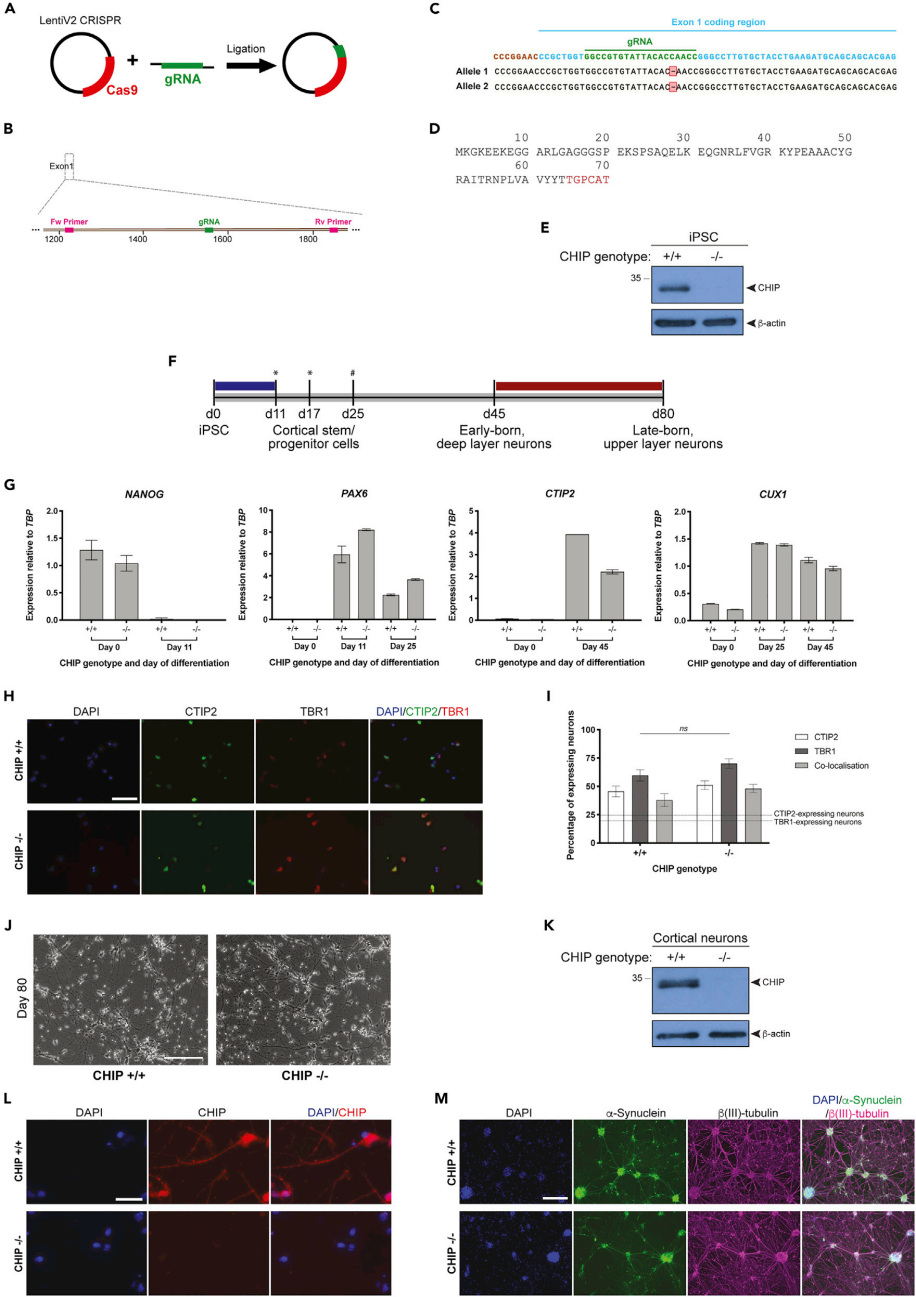
Isogenic CHIP patient-derived iPSC and cortical models (A) Using conventional cloning, the guide RNA (gRNA) targeting the gene of interest (*STUB1*) was inserted into the LentiV2 CRISPR vector carrying the Cas9 gene. Cells (cycling iPSC and undifferentiated SH-SY5Y) were transfected with this plasmid using nucleofection. (B) The gRNA used was designed to target a 20-nucleotide region (*in green*) within the first protein-coding exon of the *STUB1* gene, exon 1. The PCR primers used for screening purposes flanked this region (*shown in magenta*). (C) Sequencing of the PCR-amplified *STUB1* locus to which the gRNA targets revealed a single base-pair deletion (highlighted in red) in both alleles. (D) Protein sequence of the mutant CHIP (p.N65TfsX7) which would be encoded due to the occurrence of the indel. Such frameshift and nonsense mutations are predicted to result in the deletion of CHIP by nonsense mediated decay. (E) Steady state levels of CHIP protein in the isogenic CHIP patient-derived iPSC lines, detected by SDS-PAGE/immunoblot. (F) Schematic of the cortical differentiation protocol starting with iPSC and finishing at day 80 with both early- and late-born neurons formed. Colors indicate the different culturing conditions: differentiation media (N2 supplement and B27 supplement with retinoic acid) (*gray*), dual SMAD inhibition (SB and LDN) (*blue*), and neurotrophic factors (*red*). Throughout the differentiation, cells are either lifted as single cells (indicated by the hash) or as clumps (indicated by the asterisks). (G) The temporal specificity of the expression of different neuronal markers was assessed by qRT-PCR (normalized by TATA binding box protein (*TBP*) gene expression) in our isogenic CHIP model throughout the differentiation process. First, pluripotency markers (*NANOG* is shown) decline by day 11, while markers of cortical stem and progenitor cells (including the neuroectodermal marker, *PAX6*) increase. Once cultures are more mature, markers of both deep-layer (*CTIP2*) and upper-layer (*CUX1*) cortical neurons can be detected. Bars represent averages and error bars represent standard deviations of three technical replicates. Results are representative of two independent cortical differentiations. (H) Representative immunofluorescent images showing the expression of CTIP2 and TBR1, markers of early born, deep-layer neurons at day 50 of differentiation in CHIP KO and WT cultures. Scale bar, 100 μm. (I) Corticothalamic projection neurons of layer 6 express both TBR1 and CTIP2, while corticospinal motor neurons of layer 5 only express CTIP2. To assess the identity of the cultures, neurons expressing TBR1 and/or CTIP2 were quantified. No difference was detected in neuronal subpopulations across CHIP genotypes (Student's *t*-test) and the percentage of deep-layer neurons obtained exceeds that reported by (Shi et al., 2012b) (indicated by the dashed lines). Error bars correspond to SD. (J) Phase contrast images of the isogenic CHIP cortical model at the end of the differentiation process (day 80). The morphology and confluency across cell lines is highly similar. Scale bars, 200 μm. (K) Steady state levels of CHIP protein in the isogenic CHIP cortical model, detected by SDS-PAGE/immunoblot. (L) Representative immunofluorescent images showing the expression of CHIP in mature CHIP KO and WT-expressing cortical neurons (at day 80 of differentiation). Scale bar, 100 μm. (M) Representative immunofluorescent images showing the expression of α-synuclein and β(III)-tubulin in mature CHIP KO and WT-expressing cortical neurons (at day 80 of differentiate on). Scale bar, 100 μm.

Genetic manipulation of *STUB1* was conducted using the CRISPR/Cas9 system. To increase the likelihood of an indel knocking out the CHIP gene, a Cas9 and guide RNA (gRNA)-expressing plasmid was designed to target downstream of the start codon within the first exon of *STUB1* (Figure 1A). AST23 iPSCs were transfected by electroporation and subsequent viable single-cell colonies were validated by quantitative PCR (qPCR) (using primers flanking the gRNA as illustrated in Figure 1B). Both the PCR products and TOPO cloning products presented only one sequence, bearing a single base-pair deletion (Figure 1C). In support of both alleles of the potential CHIP KO clone having identical indels, the T7 endonuclease I assay failed to recognize and cleave non-homologous dsDNA (Figure S1). This high precision in DNA editing, driven by intrinsic preference for specific indels at target sites, is statistically favored by the presence of the adenine at position −4 from the PAM sequence in the gRNA design, and the associated strong bias toward single-nucleotide indels (Chakrabarti et al., 2019). The identical biallelic single base-pair deletion is predicted to cause a frameshift mutation, leading to a premature stop codon (Figure 1D); the transcript would be processed by nonsense-mediated decay, abolishing CHIP expression. In fact, the expression of CHIP protein in this KO clone was below the level of detection by immunoblot (Figure 1E). The iPSC AST23 cell panel therefore provide isogenic CHIP ^−/−^ and CHIP ^+/+^ (both the parental cells — referred to as P — and a control CHIP WT line that has been through the gene-editing protocol) cell models.

Moving toward a more disease-relevant model, the isogenic iPSC AST23 lines (CHIP^−/-^ plus P and WT CHIP^+/+^ controls) were differentiated into cortical neurons using an 80-day protocol that was optimized in-house. Neural induction from iPSC is triggered by a dual SMAD inhibition (Chambers et al., 2009) and is followed by cortical neurogenesis to form the six layers of the human cortex (Shi et al., 2012a, 2012b). A schematic of the cortical differentiation is shown in Figure 1F, and the appearance of different populations of cells was monitored by quantitative real-time PCR (qRT-PCR) and immunofluorescence (IF), as indicated. As expected, we observe a decline in pluripotency and stem-renewal markers (*CDK*, *NANOG*, *OCT4*) (Figures 1G and S2A) and a rise in markers of the primary cortical stem and progenitor cells (*VIM*, *NES*, *OTX2*, *EMX1,* and *PAX6*) from day 0 to day 11 (Figures 1G, S2B, and S2C). This was followed by a rise in markers of the secondary progenitor cells (including *FOXG1*) and of early born, upper-layer neurons (*CTIP2*) and late-born, deep-layer neurons (*CUX1* and *BRN2*) (Figures 1G and S2D). Fundamental principles of neural development govern the ability of primary cortical stem and progenitor cells and secondary progenitor cells to generate complex populations of cortical projection neurons, in a cell-intrinsic manner (Shi et al., 2012a). At day 50, early born neurons (TBR1 and/or CTIP2) are predominant in cultures (Figures 1H and 1I) and these persist until day 80 (Figure S2E), when neurons were harvested for proteomic analysis.

During cortical differentiation, cells are subjected to multiple morphogenic factors and undergo substantial changes in gene-expression and cell identity. It is therefore not surprising that differentiation is associated with some degree of variability, as cell-intrinsic differences influence the cellular response to the culturing conditions. However, close monitoring of cell morphology and expression profile throughout the course of differentiation support a high degree of similarity across the cell lines of interest (Figures 1G–1J and S2). This suggests that the CHIP^+/+^ and CHIP^−/-^ iPSC lines have similar differentiation potentials and dynamics, which are key criteria to allow adequate comparative proteomics.

Prior to the proteomic analyses, we confirmed that once differentiated isogenic-lines maintained differential CHIP expression profiles (Figures 1K and 1L) in an elevated α-synuclein background (Figure 1M). While the expression of *SNCA* increases throughout the differentiation with the development of neural processes and synapses, *STUB1* expression remains largely unchanged (Figure S3).

### Proteomic analysis of the isogenic cortical neurons

**Figure 2:**
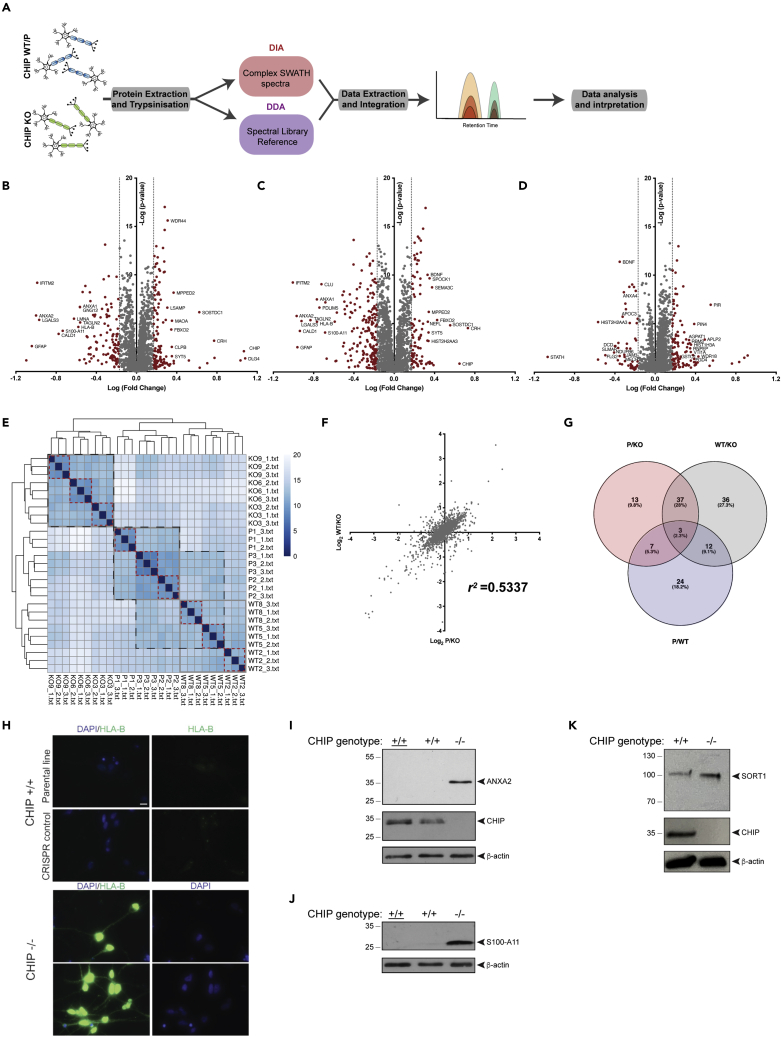
Effect of CHIP expression on the proteome of patient-derived cortical neurons (A) Rational of SWATH-MS analysis of CHIP-expressing cortical neurons (the CRISPR/Cas9 control, WT, and unedited parental line, P) and CHIP KO cortical neurons. (B–D) SWATH-MS analyses of the CHIP cortical neuronal model: P/KO (B), WT/KO (C), and P/WT (D). Cutoff criteria (≤0.67 and ≥1.5 fold change) are represented by the dashed lines. Proteins that do not meet this fold change criteria are color-coded in gray and does that do are in red. Over-represented proteins in the CHIP KO sample compared with WT have negative log (fold change), while under-represented proteins have positive values. The top 11 most under- and over-represented proteins that were significantly changed (p < 0.05) are annotated. (E) Correlation heatmap of the SWATH-MS analysis of CHIP-expressing cortical neurons (P and WT) and CHIP KO cortical neurons. All biological triplicates and technical replicates are included. Biological replicates (denoted as P1-3, WT2, 5, 8 and KO 3, 6, 9 and indicated by the three big squares outlined with gray) correlate with each other (in particular the KO replicates, P2 with P3 and WT2 with WT5), supporting the existence of limited intra-group variability. Likewise, technical triplicates (denoted as “1, 2 and 3”) show a very high correlation within each biological replicate (*squares with red outline*). The two areas outlined in black highlight high correlation across biological samples (i.e. all KO samples and most WT and P samples). (F) Correlation of the comparative proteomics analyses of CHIP-expressing cortical neurons versus CHIP KO cortical neurons (P/KO and WT/KO). Log_2_ fold changes of proteins identified in both SWATH-MS experiments are plotted. The Pearson correlation: r^2^ = 0.5337. (G) Overlap of significantly changing proteins (with fold changes ≤0.5 and ≥2) identified in the different comparative proteomics analyses, both across neuronal cultures of the same CHIP genotype (P/WT in *blue*), and different CHIP genotypes (P/KO and WT/KO, in *red* and *gray*). (H) HLA-B expression in cortical neurons of different CHIP genotypes. Nuclei were stained with DAPI. Images are z-projections (maximal projection) from stacks. Scale bar, 10 μm. (I) Steady state levels of ANXA2 protein in the isogenic CHIP cortical model, detected by SDS-PAGE/immunoblot. (J) Steady state levels of S100A11 protein in the isogenic CHIP cortical model, detected by SDS-PAGE/immunoblot. (K) Steady state levels of SORT1 protein in the isogenic CHIP cortical model, detected by SDS-PAGE/immunoblot.

Once mature (day 80 of the differentiation), cortical neurons were harvested for proteomic analysis using SWATH-MS (Figure 2A). Since it has been shown that differences in confluency can influence the proteome (Way et al., 2016), cell density was kept consistent between the cell lines at all stages of differentiation (Figures S4 and 1J). Nonetheless, in an effort to consider any differences in differentiation status, neurons derived from both P and WT control lines (CHIP^+/+^) were analyzed in comparison to the CHIP KO cells (P/KO and WT/KO, Figures 2B and 2C, respectively), as well as between each other (P/WT, Figure 2D). For each cell line, three biological replicates were analyzed and for each, technical triplicates were performed. The most over- and under-represented proteins in each comparative analysis are detailed in Tables S1–S3.

The correlation heatmap illustrates how the proteomic data sets derived from CHIP-expressing neurons (P and WT) are similar to each other and distinct from the proteome of CHIP KO neurons (Figure 2E, *areas outlined in black*). There is limited intra-group heterogeneity (with regards to biological and technical replicates – highlighted by squares outlined in gray and red, respectively). The difference across CHIP genotypes is pronounced, supporting the existence of genotype-specific proteomic changes between these groups. Beyond the expected positive linear relationship between P/KO and WT/KO (Pearson correlation index: r = 0.7305 and r^2^ = 0.5337, p value <0.0001), a subset of proteins identified were consistently under- and over-represented. These proteins are more likely to be affected, either directly or indirectly, by CHIP status and are less likely to arise from CHIP-independent factors (e.g. slight differences in differentiation status). Over- or under-represented proteins are of particular interest when they are minimally changed in the P/WT analysis or not detected at all. Of all proteins quantified by the SWATH-MS, when focusing on the significantly changed proteins only a small fraction (37 proteins) was consistently influenced by the CHIP genotype (Figure 2G and listed in Table S4).

Considering that over- and under-representation of an ionized peptide detected by MS may not directly correlate with increased or decreased protein steady state levels at a cellular level, being influenced by factors including post-translational modifications and differential ionization potential of proteins, validation is generally recommended. Immunoblot analysis of a selection of proteins from the most significantly altered list supports a linear correlation with MS identification. Hence, ANXA2, S100A11, HLA-B, and SORT1 were all upregulated in whole cell lysates from CHIP^-^^/-^ cortical neurons when compared to WT cells (Figures 2H–2K). In addition, of the proteins which meet our ≤0.5 and ≥ 2-fold change criterion, both Galectin-1 (0.38-fold) and FLNA (0.36-fold) are validated CHIP substrates (Paul and Ghosh, 2015; Wang et al., 2020). Likewise, previous proteomics identified ANXA5 (0.45-fold), HLA-B (0.22-fold) — as well as, FLNA (that here was below the level of detection by immunoblot but significantly changed in the MS analysis) — as putative CHIP substrates using an orthogonal substrate identification approach (Bhuripanyo et al., 2018). Additionally, AHNAK (0.33-fold) is present in proteomics focused on the CHIP-interactome (Kopp et al., 2017). (Note that all fold changes stated are derived from our proteomic screen).

### Pathway analysis of the CHIP cortical model

**Figure 3:**
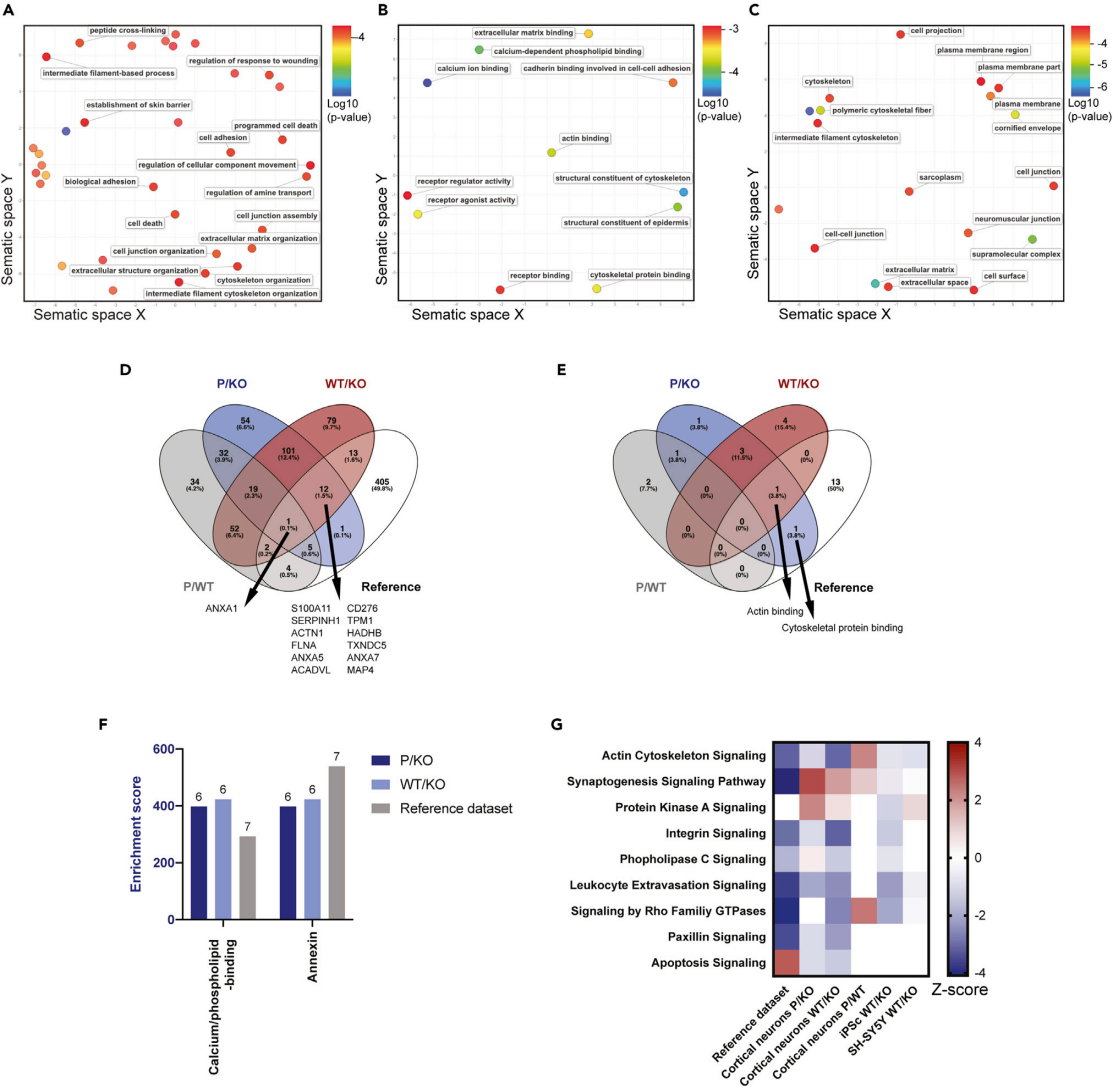
Pathway analysis of the proteome of the neuronal CHIP model and its similarities to other CHIP cell models and a model of membrane injury (A–C) The proteomic changes between CHIP-expressing cortical neurons and CHIP KO cortical neurons (P/KO and WT/KO) were analyzed by GOrilla. GO terms derived were were plotted by REduce + visualize Gene Ontology (REVIGO). GO terms shown are related biological processes (A), molecular functions (B), and cellular components (C), and each term is color-coded based on statistical significance (log10(p value)). For GO terms found in both P/KO and WT/KO, averages of the p values were considered. (D) Proteomic changes of the neuronal CHIP model share similarities with a cell model of membrane injury. Overlap of significantly changing proteins (with fold changes ≤0.67 and ≥1.50) identified in the comparative proteomics analyses of the neuronal CHIP model (both across neuronal cultures of the same CHIP genotype (P/WT), and different CHIP genotypes (P/KO and WT/KO)) and that of the injury model (annotated as “reference”). Twelve proteins were found in common in the analyses across different CHIP genotypes and the reference data set. (E) Overlap of the biological processes identified from the different comparative proteomics analyses (P/WT, P/KO, and WT/KO) and those identified from the reference data set. Data were derived from the GOrilla pathway analysis. Two processes ('calcium-dependent phospholipid binding' and 'actin binding') were identified in common from the analyses across different CHIP genotypes and the reference data set. (Additional information regarding p values and enrichment scores for each GO term, as well as the GO terms not included in the figure can be found in Table S5). (F) Gene ontology analysis by STRING of the comparative proteomics of neuronal CHIP models (WT/KO and P/KO) and the reference data set revealed similarities in molecular functions namely, 'calcium/phospholipid binding' and 'annexin'. (G) Comparative pathway analysis of the comparative proteomics of multiple CHIP cell models and the membrane injury cell model (reference data set), using Ingenuity Pathway Analysis (IPA). The heatmap shows the pathways that are most common across the data sets (-log(p value) > 1.3), along with their *Z* score, which reflects the predicted activation state of the predicted pathway (a positive/negative *Z* score indicates activation/inhibition). Results are derived from proteomic analyses of biological triplicates (for all experimental setups of cortical neurons and iPSC) or duplicates (SH-SY5Y).

To decipher cellular functions that may be regulated by CHIP and how the loss of CHIP changes the steady-state proteome, the gene ontology (GO) terms associated with the P/KO and WT/KO SWATH-MS data were defined. Firstly, we used GOrilla software (Eden et al., 2007, 2009) to obtain GO term IDs and p values that reflect the probability of their association to the P/KO and WT/KO SWATH-MS data sets. Then, significantly associated GO terms (p value < 0.05) derived from either P/KO or WT/KO SWATH-MS data sets were graphically represented using REduce + VIsualize Gene Ontology (REVIGO) (Supek et al., 2011). Interestingly, the majority of the GO terms associated to biological function (Figure 3A), molecular function (Figure 3B) and cellular component (Figure 3C) could be described as cytoskeletal- and/or membrane-related. GO terms shared between P/KO and WT/KO analyses include “cell adhesion”, “biological adhesion”, “actin binding” and “calcium-dependent phospholipid binding”. Strikingly, “actin binding” and “calcium-dependent phospholipid binding” were unique to the proteomics comparing CHIP genotype and were not found in the P/WT analysis.

Different pathway analysis software use different statistical models and reference databases. Therefore, we sought to enrich our research by incorporating other GO software, including Search Tool for the Retrieval of Interacting Genes/Proteins (STRING) (Szklarczyk et al., 2019) and Database for Annotation, Visualization, and Integrated Discovery (DAVID) (Huang et al., 2009a, 2009b). The underlying rational is that if a particular GO term is identified by multiple softwares, it is more likely to be of biological interest. Likewise, GO terms associated to the P/KO and WT/KO SWATH-MS data set and not to P/WT are of interest. These are listed in Table S5.

### Protein changes identified in CHIP cortical model are unlikely to reflect general defects in protein quality control

Given the role of CHIP in proteostasis (Connell et al., 2001; Dai et al., 2003; Kevei et al., 2017; Marques et al., 2006; Murata et al., 2001; Qian et al., 2006; Rosser et al., 2007; Zhang et al., 2015), the impact of CHIP loss-of-function on proteins involved in the regulation of the protein triage was assessed, including proteins involved in the ubiquitination system (Table S6) and molecular chaperones (Table S7). These were largely unchanged in P/KO, WT/KO, and P/WT SWATH-MS analyses, arguing against the activation of compensatory mechanisms due to the absence of CHIP expression. This is consistent with data from CHIP^−/-^ mouse cells (Morishima et al., 2008) and with a study, published during preparation of this manuscript, showing that CHIP KO neurons (at day 36) do not have an impaired heat shock response (Schuster et al., 2020). Notably, the only detected HSP that met the criterion for significant fold-change was HSP27 (*HSPB1*). HSP27 is a known regulator of actin filament stability (Doshi et al., 2009; Miron et al., 1991) and was upregulated in KO neurons. Likewise, the only protein involved in the ubiquitin system (Table S6) that was significantly changed was FAF-X (USP9X) which has been implicated in both Parkinson's- and Alzheimer's-disease (Murtaza et al., 2015). The data supports the pathway mapping and suggests that the loss of CHIP function may generate a specific cytoskeleton-related stress rather than a more general proteotoxic stress phenotype.

### Membrane- and actin cytoskeletal-related proteomics in different CHIP models

Since multiple membrane- and cytoskeletal-related GO terms were consistently identified when comparing the isogenic WT, P, and KO CHIP cell panel, we proceeded to determine whether regulation of membrane and cytoskeletal dynamics could be part of the cellular functions of CHIP. To begin, the SWATH-MS data obtained from the cortical neurons was compared to a reference data set derived from a model of cell membrane injury (Sønder et al., 2019) associated with pronounced membrane- and cytoskeleton-related changes. Twelve proteins were found to be significantly changed in both the SWATH-MS data comparing CHIP-expressing neurons to CHIP KO neurons and in the reference dataset, but excluded from the P/WT comparison (Figure 3D). With regards to the shared GO terms, “actin binding” and “cytoskeletal protein binding” were identified using GOrilla (Figures 3E and Table S8) and “calcium/phospholipid-binding” and “annexin” using STRING (Figure 3F). Therefore, the CHIP KO cortical neurons and the reference dataset share some proteomic changes, adding confidence to the observed membrane- and cytoskeletal-related proteomic changes driven by loss of CHIP function.

We reason that consistently changed processes and pathways across multiple CHIP KO models are most likely to have biological relevance. Hence, we compared the MS-based proteomics data of the isogenic CHIP cortical neuron model (Figures 2B–2D) with that of other CHIP models engineered in-house. This includes the isogenic iPSC (Figure S5) and SH-SY5Y cells (SWATH-MS data deposited in the ProteomeXchange Consortium via PRIDE: PXD016299). While the former are undifferentiated, the SH-SY5Y cells are neuronal-like and derived from neuroblastoma (Xicoy et al., 2017). The comparative pathway analysis was conducted using the IPA software (Figure 3G).

Although synaptogenesis signaling pathway and actin cytoskeleton pathway have significantly changed z-scores in all data sets of interest, only the actin cytoskeleton pathway is consistently associated with a negative *Z* score in all CHIP-expressing versus CHIP KO models (P/KO and WT/KO). Of interest, the proteomic changes observed in the reference dataset follow the same trend as the CHIP-expressing versus KO proteomics, while the proteomic analysis of CHIP-expressing cortical neurons (P/WT) shows the opposite trend. Hence, we hypothesize that CHIP influences the proteome related to the actin cytoskeleton pathway.

### Membrane protein changes identified in CHIP cortical model are not due to general defects in membrane quality control

**Figure 4:**
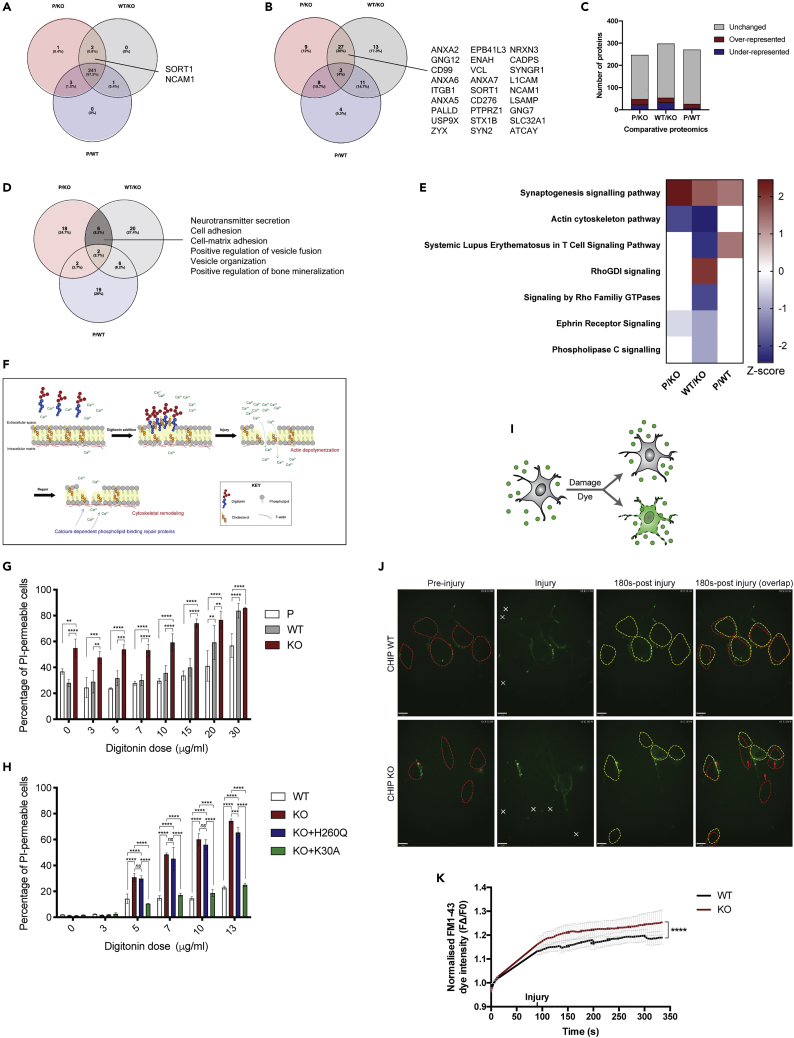
Changes in membrane proteins and actin cytoskeleton dynamics across CHIP models (A) Membrane proteins identified in the comparative proteomics analyses of the neuronal CHIP model were sorted by IPA. The vast majority were similar across all comparisons, with only two protein (SORT1 and NCAM1) being consistently identified in the P/KO and WT/KO comparisons and excluded from the P/WT analysis. (B) Significantly changed membrane proteins (with fold changes ≤0.67 and ≥1.50) found in the P/KO and WT/KO analyses and excluded from the P/WT analysis. (C) The membrane proteins identified in the SWATH-MS analyses of the neuronal CHIP model and sorted by IPA were grouped into unchanged and significantly changed (under- and over-represented when having a fold change ≤0.67 and ≥1.50, respectively). (D) Overlap of biological processes associated to the significantly changed membrane proteins across the isogenic CHIP neuronal model. Pathway analysis was conducted using DAVID. Statistical details of the GO terms associated to significant membrane proteins identified in both P/KO and WT/KO in Table S7. (E) Heatmap showing the signaling cascades predicted by the IPA to be the most significantly changed across neuronal model with differential CHIP expression. Significantly associated pathways (-log(p value) > 1.3) have a *Z* score that reflects their predicted activation state (a positive/negative *Z* score indicates activation/inhibition, respectively). (F) Schematic of the mode of action of digitonin-induced membrane injury at cholesterol-rich regions. Upon injury and the subsequent influx of calcium, actin becomes depolarized. This triggers the recruitment of calcium-dependent phospholipid-binding repair proteins and induces substantial cytoskeletal remodeling for repair. (G and H) iPSC (G) and undifferentiated SH-SY5Y (H) of different CHIP genotypes were incubated with increasing doses of digitonin. Other than CHIP KO cells and those expressing WT CHIP, undifferentiated SH-SY5Y expressing the E3 ligase-dead CHIP mutant (H260Q) or the chaperone-dead CHIP mutant (K30A) were also included. Injured and control cells were incubated with PI and cell-permeable Hoechst, allowing quantification of PI^+^ cells in a population of cells (reflective of increased permeability). Three biological replicates were included per condition and cell line. Bars represent mean ± SD. two-way ANOVA, p < 0.05, ∗∗p < 0.005, ∗∗∗p < 0.0005, ∗∗∗∗p < 0.0001. (I) Schematic of the repair kinetics assay using UV laser-induced injury and a cell impermeable dye, FM1-43. Damage to the cell membrane results in a gradual intracellular accumulation of FM1-43 dye. (J) UV laser-induced injury (represented by the crosses) caused undifferentiated SH-SY5Y cells to incorporate the cell impermeable dye FM1-43 in the cell bodies in a time-dependent manner. The outline of the cell bodies before (*red*) and after (*yellow*) injury are shown. Cells that have migrated in the aftermath of the injury are highlighted by the red arrows. Representative images of CHIP WT and KO cells at pre-injury, injury, and 180s-post injury are shown. Scale bar 9 μm. (K) Undifferentiated SH-SY5Y CHIP KO cells incorporate more of the FM1-43 dye than the CHIP KO cells. The fluorescence intensity (F) within cell bodies was quantified over time and normalized by the pre-injury fluorescence (FΔ/F0). Averages were plotted. Student's *t*-test, ∗∗∗∗p < 0.0001, n ≥ 16 cell bodies.

Cytoskeletal-based processes are intimately connected to membrane proteins (Bezanilla et al., 2015; Boucher and Mandato, 2015; Fais et al., 2000; Gov, 2018; Jaiswal et al., 2014; Sønder et al., 2019). We therefore questioned whether the changes observed were due to a broad effect on bulk membrane protein homeostasis, or whether there was a more targeted effect of CHIP loss on a subset of membrane-associated proteins. Accordingly, we first sorted all cell membrane proteins identified across all SWATH-MS comparisons conducted using IPA. The vast majority of membrane proteins identified were similar across all comparisons, with only two proteins (SORT1 and NCAM1) being consistently identified in the P/KO and WT/KO comparisons and excluded from the P/WT analysis (Figure 4A). Likewise, when focusing on membrane proteins with significant fold-changes, most were found in the P/KO and WT/KO analyses and excluded from the P/WT analysis (Figure 4B). However, the proportion of significantly changed to unchanged membrane proteins was similar throughout all comparisons (Figure 4C). The data therefore suggest that, whilst CHIP loss does not affect a broad cross-section of membrane proteins, it does impact on a focused cohort of membrane associated proteins. The membrane subcellular localization of the INSR has previously been shown to be regulated by CHIP, through ubiquitination (Tawo et al., 2017). Accordingly, the steady state levels of the INSR are increased in CHIP KO neuronal and SH-SY5Y models (Figure S6). This effect of CHIP in the regulation of membrane proteostasis is associated with its role in mediating longevity.

To assess the specific membrane process that could be impacted by loss of CHIP function, the biological processes related to these membrane proteins (Figures 4A and 4B) were identified using DAVID (Figure 4D and Table S9). Processes that are unlikely to be CHIP-dependent are those only found in the P/WT comparison, while processes more likely to be CHIP-dependent are exclusively derived from both the P/KO and WT/KO analyses. Only a minority (8%) of biological processes fell in the latter group (*highlighted in dark gray in* Figure 4D), and most of these are related to cell adhesion and the regulation of vesicles — processes that rely on cytoskeletal architecture. Furthermore, actin cytoskeleton was the only canonical pathway predicted by the IPA software that was consistently and significantly changed in P/KO and WT/KO analyses and not in the P/WT comparison (Figure 4E).

### CHIP KO cells have a compromised membrane integrity

We reasoned that if the CHIP-mediated membrane- and cytoskeletal-related proteomic changes described above reflected differences in biology, a measurable phenotypic change should be detected in the KO cells compared with controls. To elucidate any underlying differences, membrane and actin cytoskeleton remodeling events were induced by membrane injury. It has been shown that extensive actin de- and re-polymerization occurs upon membrane damage, in addition to the recruitment of several cytoskeletal and membrane-binding proteins (Boucher et al., 2019; Boucher and Mandato, 2015; Jaiswal et al., 2014; Simonsen et al., 2020). Membrane injury was inflicted using digitonin (an inducer of pores in cholesterol-rich regions of the plasma membrane — illustrated in Figure 4F) and monitored by the intracellular leakage of a membrane-impermeable dye (propidium iodide, PI) (Jaiswal et al., 2014). Here, PI is not only a marker of membrane integrity but also of cell death (Cummings et al., 2012).

Prior to performing damage experiments in isogenic SH-SY5Y cells, we first established if the protein expression changes seen in CHIP KO neurons were reproduced in the neuroblastoma background. Whereas S100A11 did not increase in either the MS analysis or the immunoblots in response to the loss of CHIP in SH-SY5Y cells (Figure S7A), the steady state levels of both ANXA2 and SORT1 where elevate in this cell background (compared to the isogenic WT, Figures S7B and S7C). The accumulation of ANXA2 and SORT1 in two independent CHIP KO cell models of neuronal origin suggests that they may be core components of the CHIP-regulated proteome in neuronal cells.

Following exposure to digitonin both CHIP KO iPSC (Figure 4G) and SH-SY5Y (Figure 4H), cells were consistently found to accumulate intracellular PI to a greater extent than CHIP-expressing cells. The result suggests increased membrane “leakiness” and sensitivity to membrane damage in both cell lines. CHIP KO iPSC also incorporated more intracellular PI than the controls prior to injury, highlighting potential differences in membrane integrity under basal conditions. CHIP KO SH-SY5Y cells were rescued by the stable expression of a K30A mutant CHIP (Figure 4H) – this mutant no longer binds HSP70 but retains some E3-ligase activity (Narayan et al., 2015). In contrast, no rescue was seen with an E3-ligase dead mutant of CHIP (H260Q; Figure 4H). The latter result supports a specific role for the E3-ligase function of CHIP in membrane integrity.

Membrane injury was next induced by a UV-ablation laser and monitored by live-imaging using a membrane-impermeable dye FM1-43 (Jaiswal et al., 2014) added to the culture media (Figure 4I). Supporting the digitonin data, CHIP KO SH-SY5Y cells incorporated more FM1-43 dye than WT cells following laser-induced injury (Figures 4J and 4K). Of note were differences in the cell morphology and behavior upon injury: CHIP KO cells migrated more, retracted their projections and adopted a rounded phenotype post-injury, while WT cells remained largely unchanged post-injury (Figure 4J).

Together, phenotypic data reveal differences in membrane and cytoskeletal homeostasis in the absence of CHIP function. At a cellular level, CHIP KO cells have a compromised membrane integrity, suffering from increased permeability under basal conditions and have increased susceptibility to membrane damage upon injury.

## Discussion

Until relatively recently the prevailing hypothesized for the function of the CHIP E3-ligase has almost been exclusively explained through its ability to bind core components of the molecular chaperone machinery and, in doing so, actively take part in the degradation of HSP-client proteins (Dickey et al., 2007; Joshi et al., 2016; Kampinga et al., 2003; Marques et al., 2006; Murata et al., 2001; Paul and Ghosh, 2015; Zhang et al., 2005). More than 226 diverse putative substrates have been identified for CHIP (Bhuripanyo et al., 2018; Joshi et al., 2016; Marques et al., 2006), with the vast majority linked to HSP70-binding and substrate degradation. This view was supported by a recent study comparing iPSC-derived neurons from SCAR16 patients (with various *STUB1* mutations) to those from healthy individuals. The proteomic changes identified in the Schuster et al., 2020 study were primarily associated with oxidative and proteotoxic stress. In the current study we were able to uncouple the role of CHIP in oxidative stress and protein quality control (QC) from its non-canonical activities by using isogenic cells in an early stage disease background. Our aim was to identify processes that are involved in CHIP-dependent neuroprotection at early disease stages, when wild-type CHIP is still active, in expectation that these become less significant at later disease stages and disease progression is favored, for example when CHIP activity is downregulated due to sequestration into Lewy bodies (Shin et al., 2005). What we found was that loss of CHIP function in the AST23 background produced detectable steady state changes in a relatively focused cohort of proteins (Figures 2B and 2C). Proteins that were most significantly changed place the ligase as a determinant of actin cytoskeleton function and homeostasis of specific membrane proteins. A role for CHIP in membrane biology is of interest as membrane homeostasis has recently become a subject of investigation in α-synucleinopathies (Bozelli et al., 2021; Dettmer et al., 2017; Rovere et al., 2019) and, more generally, in neurodegenerative conditions (Bezprozvanny and Hiesinger, 2013; de Groot and Burgas, 2015; Marin, 2013).

At the single cell level, the repair of membrane structural damage is an active process that requires the coordinated manipulation of both the cell's cytoskeletal and membrane compartments (Boucher et al., 2019; Boucher and Mandato, 2015). This is evident from the proteomics analysis of the reference database a comparative proteomic analysis of a cell model of membrane injury described previously (Sønder et al., 2019). Of note, proteins with membrane- and cytoskeletal-binding properties were not only found to be significantly enriched in this data set (Figures 3E and 3F) but also across all of the CHIP WT/KO models (Figure 3G). Proteins significantly changed both in response to CHIP loss in neuronal cells and to membrane injury (the reference database) belong either to the membrane repair protein machinery (including ANXA5 (Bouter et al., 2011) and ANXA7 (Sønder et al., 2019)) or the cytoskeleton network (ACTN1 (Naumanen et al., 2008), FLNA (Yue et al., 2013), TPM1 (Janco et al., 2016), and MAP4 (Kitazawa et al., 2000)) (Figure 3D). Similarities in proteomic changes derived from loss of CHIP and exposure to membrane stress opens the question whether altered membrane homeostasis may be of relevance in human diseases associated to CHIP loss-of-function.

Membrane injury triggers extensive cytoskeletal remodeling. Specifically, the resultant calcium influx drives actin remodeling (Boucher and Mandato, 2015), protein-driven modulation of the membrane and the recruitment of cytoskeletal-remodeling proteins to facilitate wound closure (Boye et al., 2018; Jaiswal et al., 2014; Simonsen et al., 2020). Apart from its role in modulating membrane tension, cytoskeleton remodeling is also required for endocytosis- and exocytosis-mediated repair (Boucher and Mandato, 2015; Cooper and McNeil, 2015). The integrity of the cytoskeleton and its ability to reorganize rapidly in response to external and internal stimuli is key for normal cell function (Kaminska et al., 2011). It has been reported that E3 ligases can modulate actin cytoskeleton dynamics (reviewed in (MacGurn et al., 2012), these include NEDD4 (Stawiecka-Mirota et al., 2008), MURF2 (Silvestre et al., 2019), RSP5 (Kaminska et al., 2011), OZZ (Bongiovanni et al., 2012), and CLB (Watanabe et al., 2013). Although the role of the ubiquitin-proteasome system in membrane repair via modulation of the cytoskeleton has not been investigated extensively, it has been proposed (Cai et al., 2009; Ibañez-Vega et al., 2019; Watanabe et al., 2013). For example, ubiquitin-dependent, chaperone-assisted selective autophagy (CASA) may be related to membrane fragility and to the cytoskeletal changes observed in our model due to the absence of CHIP. CASA is a BAG3-dependent process that recruits HSP70 and CHIP to form active complexes (Arndt et al., 2010). FLNA, an actin and membrane protein cross-linker, when released from actin filaments after rounds of muscle contraction is degraded in the lysosomes via CASA. Interestingly, FLNA has been previously validated as a CHIP substrate (Arndt et al., 2010; Paul and Ghosh, 2015) and was identified in our data set. Although a mutant of CHIP (K30A), which cannot bind to HSP70 (Narayan et al., 2015), complements the membrane-damage phenotype in CHIP KO SH-SY5Y cells (Figure 4H), we cannot rule out a role for CASA in explaining the molecular and cellular phenotype observed, as the precise architecture of the complex and the mechanism of CHIP recruitment remains to be determined.

Membrane protein homeostasis is of utmost importance to maintain membrane integrity and cell function (Kevei et al., 2017). Plasma membrane QC mechanisms are not only employed during membrane “remodeling” in the aftermath of injury (to remove damaged components and restore adequate protein) (Boucher and Mandato, 2015; Cooper and McNeil, 2015; Davenport et al., 2016; Häger and Nylandsted, 2019; Moe et al., 2015) but also as part of housekeeping functions under basal conditions (Meacham et al., 2001; Morishima et al., 2008; Tawo et al., 2017; Zhao et al., 2013). Although the underlying QC mechanisms for the membrane are still largely unknown, ubiquitination-based mechanisms have been proposed (MacGurn et al., 2012). Accordingly, there is growing evidence for the role of CHIP in membrane protein biology, such as in the regulation of the misfolded fibrosis transmembrane-conductance regulator (CFTR) (Meacham et al., 2001). CFTR regulation is part of the canonical functions of CHIP, being HSP-dependent (Okiyoneda et al., 2010). However, more recent evidence points to a role for CHIP in the regulation of folded membrane proteins, including GR (Morishima et al., 2008) and INSR (Tawo et al., 2017). The latter, however, only occurs in the absence of cellular stress (Tawo et al., 2017). Also recently, it has been shown that CHIP can be transiently recruited to cellular membranes, having affinity for specific phospholipid species (Kopp et al., 2017). CHIP localization to membranes is shown to be HSP70-independent, again pointing to a non-canonical function for CHIP in membrane protein regulation.

Here, we found that loss of CHIP function in neuronal cells leads to changes in the MS-profile that were indicative of steady state changes in a specific subset of membrane-associated proteins (Figure 4B), whereas the bulk of membrane proteins were unaffected by loss of CHIP (Figure 4C). The data therefore suggest a role for CHIP in the regulation of specific membrane components rather than a general loss of integrity due to more widespread defects in membrane QC. Interestingly, the majority of the membrane proteins influenced by CHIP status are involved in maintaining membrane integrity, having established roles in membrane repair (ANXA2, ANXA6, ANXA5, ANXA7 (Draeger et al., 2011) and Zyxin (Lecroisey et al., 2013)) — or a putative role in exocytosis-mediated membrane repair (Borgonovo et al., 2002; Cai et al., 2009; Cooper and McNeil, 2015), namely CADPS (Cisternas et al., 2003) and SYNGR1 (Sugita et al., 1999) — and in regulation of the actin-cytoskeleton (ITGB1 (Otey et al., 1990), PALLD (Yadav et al., 2016), EPB41L3 (Baines et al., 2014), ENAH (Krause et al., 2003), VCL (Le Clainche et al., 2010), and SYN2 (Bloom et al., 2003) (Figure 4B). Thus, by controlling membrane properties (directly or indirectly), CHIP may influence cytoskeleton and membrane dynamics. This is illustrated by the finding that CHIP KO cells were consistently more sensitive to membrane stressors (Figures 4G–4K) and had defective repair kinetics (Figure 4K). The induction of membrane stress revealed a significant phenotypic consequence of loss of CHIP function. A president for how E3-ligases can impact on membrane homeostasis can be found from studies on Rap5. Loss of Rap5 ubiquitin ligase function leads to impaired QC of membrane proteins, causing their accumulation at the plasma membrane and loss of membrane integrity (Zhao et al., 2013). However, our data suggest a role for CHIP in the regulation of specific membrane components rather than a general loss of integrity due to more widespread defects in membrane protein QC.

The intimate relationship between actin cytoskeleton and membrane dynamics is clear. However, how these are regulated to ensure adequate membrane repair responses and overall maintenance of membrane integrity and cell function is less clear. Data presented suggest that CHIP can influence actin cytoskeleton signaling and membrane integrity in different cell models, including cortical neurons. Given the growing link between plasma membrane QC, membrane integrity and neurodegeneration (Naudí et al., 2013; Shrivastava et al., 2017; Wong-Ekkabut et al., 2007; Zhao et al., 2013), as well as cytoskeletal defects and neurodegeneration (e.g. tauopathies) (Bamburg and Bloom, 2009), understanding how CHIP influences both the cytoskeleton and plasma membrane integrity could provide insights into disease mechanisms. In addition, identification of non-canonical activities of CHIP may help to explain how CHIP is neuroprotective and suggest how mutation or sequestration of CHIP impacts cognitive health.

### Limitations of the study

The amount of material we were able to generate from iPSC-derived cortical neurons was limiting. Therefore, the mass spectrometry analysis likely detected the dominant steady state changes in proteins modulated by CHIP and under-represented proteins present at very low cellular concentrations. For example, we were not able to detect IRF-1, a well characterized CHIP substrate, which is expressed at very low levels in cultured cells. Nonetheless, to limit this under-representation, analysis was conducted using the data-independent mode. In addition, our study was designed to measure changes in protein levels rather than identifying CHIP-dependent changes in the ubiquitinome. As a consequence, the proteins found to be differentially present in CHIP wild-type and KO neurons are not necessarily CHIP substrates but could be modulated indirectly by CHIP. Following on from this, CHIP substrates subjected to ubiquitination that is not linked to changes in protein level, for example, those modified with K63-linked polyubiquitin would not have been identified. The iPSC-derived cortical model used also has some inherent limitations. First, both the genetic editing and the cortical differentiation are subjected to clonal variability. Hence, multiple CHIP cell models were investigated and only consistent changes were considered. Furthermore, our isogenic CHIP iPSC panel was restricted to a single CHIP KO cell line due to the difficulties in generating viable CHIP KO clones in the AST23 background. Finally, our ability to carry-out membrane damage experiments in cortical neurons, as opposed to iPSC or SH-SY5Y cells, was limited as even the wild-type neurons were extremely sensitive to the methodologies used making well controlled experiments impractical.

## STAR★Methods

### Key Resources Table

**Table:** 

REAGENT or RESOURCE	SOURCE	IDENTIFIER
**Antibodies**		

Mouse monoclonal anti-CHIP	Vojtesek Lab (Narayan et al., 2015)	3.1
Mouse monoclonal anti-α-synuclein	BD Biosciences	610787; RRID: AB_398108
Mouse monoclonal anti-β-actin	Sigma	A5441; RRID: AB_476744
Mouse (IgG2b) monoclonal anti-β(III)-tubulin	Sigma	T8578; RRID: AB_1841228
Rat monoclonal anti-CTIP2	Abcam	ab18465; RRID: AB_2064130
Rabbit polyclonal anti-TBR1	Abcam	ab31940; RRID: AB_2200219
Mouse monoclonal anti-Pax6	DSHB	AB_528427; RRID: AB_528427
Mouse monoclonal anti-SORT1	BD Transduction Laboratories	612101/NTR3; RRID: AB_399472
Rabbit polyclonal anti-ANXA2	Abcam	ab41803; RRID: AB_940267
Rabbit polyclonal anti-S100A11	Proteintech	10237-1-ap; RRID: AB_2183478
Goat polyclonal anti-Mouse (IgG1) A488	Invitrogen	A21121; RRID: AB_2535764
Goat polyclonal anti-Mouse (IgG2b) A647	Invitrogen	A21242; RRID: AB_1500900
Goat polyclonal anti-Mouse A555	Invitrogen	A21127; RRID: AB_141596
Donkey polyclonal anti-Rat A488	Invitrogen	A21208; RRID: AB_141709
Donkey polyclonal anti-Rabbit A555	Invitrogen	A31572; RRID: AB_162543
Rabbit polyclonal horseradish peroxidase-conjugated anti-Mouse	Dako	PO260
Swine polyclonal horseradish peroxidase-conjugated anti-Rabbit	Dako	PO217

**Chemicals, Peptides, and Recombinant Proteins**		

LDN	Miltenyi Biotech	130-103-925
SB431542	Merck Chemicals, Calbiochem	616461
Y27632 dichloride	R&D, Tocris	1254
Laminin-111	Biolamina	LN111
Laminin-521	Biolamina	LN521
Digitonin	Sigma	D141
Propidium iodide	Sigma	P4864
FM1-43 dye	Invitrogen	T3163
Hoechst-33342	Invitrogen	H3570

**Critical Commercial Assays**		

Human Stem Cell Nucleofector	Lonza	VPH-5012
LightCycler® 480 UPL Probe Master	Roche	04707494001
Quantitative Colorimetric Peptide Assay	Thermo Scientific	23275
RC-DC assay	Bio-rad	5000121

**Deposited Data**		

SWATH-MS data: comparative analysis of CHIP-expressing (parental line) and CHIP KO iPSC-derived cortical neurons	This paper (Data S1)	PRIDE: PXD021404
SWATH-MS data: comparative analysis of CHIP-expressing (CRISPR/Cas9 control) and CHIP KO iPSC-derived cortical neurons	This paper (Data S2)	PRIDE: PXD021404
SWATH-MS data: comparative analysis of CHIP-expressing iPSC-derived cortical neurons (parental line and CRISPR/Cas9 control)	This paper (Data S3)	PRIDE: PXD021404
SWATH-MS data: comparative analysis of CHIP WT and CHIP KO iPSC	This paper (Data S4)	PRIDE: PXD021404
SWATH-MS data: comparative analysis of CHIP WT and CHIP KO SH-SY5Y cells	(Nita, 2016)	PRIDE: PXD016299

**Experimental Models: Cell Lines**		

AST23 iPSC: Parental line	Kunath Lab (Devine et al., 2011)	N/A
AST23 iPSC: CHIP KO	This paper	N/A
AST23 iPSC: CRISPR/Cas9 control (CHIP WT)	This paper	N/A
SH-SY5Y: Parental line	Kunath Lab (Drummond et al., 2017)	N/A
SH-SY5Y: CHIP KO	(Nita, 2016)	N/A
SH-SY5Y: CRISPR/Cas9 control (CHIP WT)		
SH-SY5Y: CHIP KO expressing wtCHIP (stable transfection)	(Nita, 2016) Plasmids used were established in (Narayan et al., 2015)	N/A
SH-SY5Y: CHIP KO expressing CHIP H260Q (stable transfection)		
SH-SY5Y: CHIP KO expressing CHIP K30A (stable transfection)		
**Oligonucleotides**		

RT-qPCR primers	This paper	Table S10
Forward *STUB1* primer AGAACGAGGGTGCGATGC	This paper	N/A
Reverse *STUB1* primer GATGTCGTCCCCGAAGTTCA	This paper	N/A

**Recombinant DNA**		

gRNA(CHIP) cloned into LentiV2 backbone	(Nita, 2016)	N/A

**Software and Algorithms**		

Prism GraphPad	GraphPad	https://www.graphpad.com/scientific-software/prism/
SnapGene	GSL Biotech LLC	https://www.snapgene.com/
MarkerView 1.2.1.1	AB-SCIEX	https://sciex.com/content/dam/SCIEX/pdf/customer-docs/release-notes/MarkerView_1.2.1_relNotes.pdf
ProteinPilot 4.5	AB-SCIEX	https://sciex.com/content/dam/SCIEX/pdf/software/ProteinPilot_Software_501_Release_Notes.pdf
SWATH™ Acquisition MicroApp 1.0 a plugin of PeakView 1.2.0.3	AB-SCIEX	https://sciex.com/content/dam/SCIEX/pdf/customer-docs/release-notes/SWATH_Processing_Release_Notes1.pdf
GOrilla	(Eden et al., 2007, 2009)	http://cbl-gorilla.cs.technion.ac.il/
REduce + VIsualize Gene Ontology (REVIGO)	(Supek et al., 2011)	http://revigo.irb.hr/
Database for Annotation, Visualization and Integrated Discovery (DAVID)	(Huang et al., 2009a; 2009b)	https://david.ncifcrf.gov/content.jsp?file=functional_annotation.html
Search Tool for the Retrieval of Interacting Genes/Proteins (STRING)	(Szklarczyk et al., 2019)	https://string-db.org/cgi/input?sessionId=bI3XHaoMFOA0&input_page_active_form=multiple_identifiers
Ingenuity Pathway Analysis (IPA)	Qiagen	https://digitalinsights.qiagen.com/products-overview/discovery-insights-portfolio/analysis-and-visualization/qiagen-ipa/?cmpid=QDI_GA_IPA&gclid=CjwKCAjw3pWDBhB3EiwAV1c5rHy2w0yPhQ699r3__ycWTHXrCPf3LBaTW4BUNfYAmpaSW2noKMgU4hoCRScQAvD_BwE
Illustrator	Adobe	https://www.adobe.com/products/illustrator.html
Volocity	Quorum Technologies	https://quorumtechnologies.com/volocity/volocity-downloads/downloads
Celigo Software Version 2.1	Nexcelom Bioscience	https://www.nexcelom.com/nexcelom-products/cellometer-and-celigo-image-cytometers/celigo-imaging-cytometer/
Micromanager Software (μManager)	Vale Lab	https://micro-manager.org/
ImageJ	(Schneider et al., 2012)	https://imagej.nih.gov/ij/download.html

### Resource availability

#### Lead contact

Further information and requests for resources and reagents should be directed to and will be fulfilled by the lead contact, Kathryn Ball (kathryn.ball@ed.ac.uk).

#### Materials availability

This study generated a CHIP KO iPSC line carrying a triplication of the *SNCA* gene (AST23-CHIPKO) and differentiated this iPSC line into mature cortical neurons. The isogenic CHIP ^-/-^ and CHIP ^+/+^ iPSC lines are available.

#### Data and code availability

The published article includes all datasets generated or analyzed during this study:

Table S1 and Data S1: The most under- and over-represented proteins (Table S1) or all proteins identified (Data S1) in the SWATH-MS analysis comparing parental cortical neurons (genetically unedited) to CHIP KO cortical neurons. Related to Figure 2.

Table S2 and Data S2: The most under- and over-represented proteins (Table S2) or all proteins identified (Data S2) in the SWATH-MS analysis comparing CHIP-expressing cortical neurons (derived from a CRISPR/Cas9 control clone) to CHIP KO cortical neurons Related to Figure 2.

Table S3 and Data S3: The most under- and over-represented proteins (Table S3) or all proteins identified (Data S3) in the SWATH-MS analysis comparing CHIP-expressing cortical neurons (the parental line and the CRISPR/Cas9 control line). Related to Figure 2.

Data S4: All proteins identified in the SWATH-MS analysis comparing CHIP-expressing iPSC (derived from a CRISPR/Cas9 control clone) to CHIP KO iPSC. Related to Figure 2.

The mass spectrometry proteomics data have been deposited to the ProteomeXchange Consortium via the PRIDE (Perez-Riverol et al., 2019) partner repository with the dataset identifier PXD021404.

### Experimental model and subject details

#### Cell lines

AST23 (female): iPSC cells were cultured on Laminin 521-coated plates (Biolamina) for routine culturing and maintained on iPS-Brew XF media (130-104-368, StemMACS, Miltenyi Biotech Inc.), 37°C and 5% CO_2_ in a humidified atmosphere. iPSC were passaged using 0.5 mM EDTA (UltraPure 0.5 M EDTA, pH 8, 15575020, Gibco), diluted in dPBS without calcium and magnesium (D8537, Sigma). This line was previously authenticated by SNP and gene expression microarray (refer to Devine et al., 2011).

SH-SY5Y (female): Human neuroblastoma cells were maintained on DMEM (41965-039, Gibco), 10% fetal bovine serum (10270-106, Gibco), 37°C and 5% CO_2_ in a humidified atmosphere. When 70-80% confluence, cells were passaged using trypsin (0.05% trypsin +0.02% EDTA, Gibco, 15090).

All cell lines were routinely tested for mycoplasma using the Luciferase-based MycoAlertTM detection kit (Lonza) and cell authentication was performed by morphology and molecular marker checking.

### Method details

#### Generation of CHIP KO iPSC using CRISPR/Cas9 technology

The gRNA was designed to target downstream of ATG within the first coding exon (exon 1) of *STUB1*, increasing the likelihood of an indel mutation knocking out CHIP protein. The guide sequence used is GGCCGTGTATTACACCAACC GGG (PAM sequence underlined). According to the MIT software, it has a quality score of 94% and predicted to have 30 off-target sites, of which 7 are located in genes. Previously, this gRNA was inserted into a LentiV2 backbone containing the Cas9 gene and successfully generated homozygous CHIP KO SH-SY5Y cells (E.N., unpublished data).

AST23 iPSC were first lifted with Accutase (A6964, Sigma) and incubated in culturing media supplemented with 10 μM Y27632 dichloride (1254, R&D, Tocris) to promote survival. Cells were transfected using the Human Stem Cell Nucleofector (VPH-5012, Lonza) according to the manufacturer’s instructions. Electroporation was performed with the B-016 program in a NucleofectorTM 2b Device (AAB-1001, Lonza). Cells were plated at low density on Laminin 521-coated plates (Biolamina) and incubated with the culturing media supplemented with 10 μM Y27632 dichloride (1254, R&D, Tocris) for two days.

Once single cells grew into small colonies they passaged onto individual wells within a 96-well plate. Prior to this, cells were treated for 2 h with culturing medium supplemented with 10 μM Y27632 dichloride. Cells were treated with 250 U/ml of Collagenase Type IV (17104019, Gibco) until colonies begin to detach. Single colonies were picked manually, as single cell sorting tends to be too stressful for iPSCs. Picked colonies were maintained in culturing medium was supplement with 0.5 U/ml Penicillin + 500 ng/ml Sreptomycin (15140122, Gibco) and 10 μM Y27632 dichloride. Once clones grew from the 96-well plate, they were passaged into incrementally increasing plates until they were validated (by immunoblotting, immunofluorescence and sequencing) and cryopreserved. Sequencing data obtained was analyzed using SnapGene to identify indels within the *STUB1* gene of clones derived from CRISPR/Cas9-based gene editing.

#### Polymerase chain reaction (PCR) genotyping

Chromosomal DNA was extracted from frozen cell pellets using the Gentra Puregene Cell kit (Qiagen) following manufacturer’s instructions. *STUB1* gene amplification was performed using the high fidelity *Pfu* DNA polymerase according to manufacturer’s instructions and both forward (AGAACGAGGGTGCGATGC) and reverse (GATGTCGTCCCCGAAGTTCA) primers. PCR cycling was performed using the Agilent SureCycler 8800 machine and DNA Sanger sequencing was conducted by Source Bioscience (LifeSciences).

#### TOPO-cloning

PCR products obtained using *Taq* DNA polymerase (M1661, Promega) were sub-cloned into a vector (pCR 2.1-TOPO TA) optimized for high efficiency transformation, using the TOPO-TA Cloning Kit for Subcloning (450641, Invitrogen) according to the manufacturer’s instructions. DH5α competent cells were transformed and the DNA of single colonies was extracted using the QIAprep Spin Miniprep Kit (27104, Qiagen) and sequenced.

#### T7 Endonuclease I assay

PCR products with *STUB1* amplified were subjected to the T7 endonuclease I assay (New England Biolabs) according to manufacturer’s instructions. The reactions were electrophoresed in a 1% agarose gel with SYBR®Safe DNA Gel Stain (Invitrogen), which was also loaded with the Quick-Load 100 bp DNA ladder (New England Bioscience).

#### Quantitative reverse transcription PCR (qRT-PCR)

RNA was isolated using the Epicentre MasterPureTM Complete DNA and RNA Purification Kit (Epicentre, MC85200) and treated with riboguard RNAse inhibitor (Invitrogen, 10777019) and DNAse I (New England Biolabs), according to the manufacturer’s instructions. cDNA was synthesized from 1 μg of DNAseI-treated RNA using the M-MLV Reverse Transcriptase (200 units/μl) (28025013, Invitrogen). RT-qPCR was performed using the Universal Probe Library (UPL) (Roche). The Roche UPL Assay design centre was used to design the primers (when available, these were intron-spanning) with specific UPL probes for each gene (Table S10). Reactions (10 μl) containing cDNA, primers, UPL probe, LightCycler® 480 UPL Probe Master mix (Roche) and PCR water were performed in 386-well plates as described by the manufacturer’s instructions. For each primer used three technical replicates were included. The data were normalized to the levels of *TATA-binding protein* (*TBP*).

#### SDS-PAGE and immunoblotting

For immunoblotting, cells were harvested by scrapping (after being washed with PBS) and pellets were lysed with 8M urea, 50 mM Hepes pH 8, 100 mM KCl, 1mM DTT and 0.2% Triton X-100. Protein concentration of lysed samples was quantified using Protein Assay Dye Reagent (Bio-Rad), according to the manufacturer’s instructions. Proteins were resolved on 12% polyacrylamide gels and transferred onto 0.2 μM nitrocellulose membranes (Amersham Protran, GE Healthcare). Immunoblots were blocked with 5% skimmed milk powder in PBS containing 0.1% Triton X-100 and then incubated with either an in-house primary antibody against CHIP, anti-ANXA2, anti-S100A11, anti-SORT1 or anti-β-actin diluted in the block solution for 1h at room temperature shaking. The secondary antibody used was anti-mouse or anti-rabbit and conjugated with horseradish peroxidase also diluted in the block solution. Immunoblots were processed by enhanced chemiluminescence (ECL).

#### Immunofluorescence

Cells were fixed with 4% PFA and blocked with 2% donkey serum (Sigma) in PBS with 0.1% Tween-20. Primary antibodies used were α-synuclein, β(III)-tubulin, CHIP, CTIP2, TBR1, Pax6 and HLA-B. Secondary antibodies used include anti-mouse A488, anti-mouse IgG2b A647, anti-mouse A555, anti-rat A488 and anti-rabbit A555, alongside DAPI. Cells were imaged using the Axio Imager (Zeiss) and the microscope hardware was controlled by Micromanager Software (μManager). ImageJ was used to create maximal projections from z-stacks of images.

#### Cortical differentiation from iPSC

The dual SMAD inhibition protocol used for neural induction was based on published protocols (Chambers et al., 2009; Shi et al., 2012a, 2012b) and optimized for our iPSC model. Cells were plated at 80,000 cells/cm^2^ on laminin 111-coated plates (Biolamina) and treated with 10 μM Y27632 dichloride (1254, R&D, Tocris) for 48h. Once seeded they were maintained in differentiation media containing DMEM/F12 (21331-020, Gibco):Neurobasal media (21103-049, Gibco) in 1:1 ratio, B27 supplement with retinoic acid (17504044, Gibco), N2 supplement (17502001, Gibco) and 2mM L-glutamine (25030-024, Gibco). For 11 days, iPSCs were treated with 10 μM SB431542 (616461, Merck Chemicals, Calbiochem) and 100 nM LDN (130-103-925, Miltenyi Biotech) and the media was changed daily.

For cortical neurogenesis, cells were passed at day 11 and plated onto laminin 111-coated plates (Biolamina) in a 1:1.5 ratio. For such, cells were incubated with Collagenase Type IV (250 U/ml, 17104019, Gibco) until they detach as a monolayer and then triturated by pipetting until small aggregates form. After washes, cells were incubated with differentiation media supplemented with 10 μM SB431542 (616461, Merck Chemicals, Calbiochem), 100 nM LDN (130-103-925, Miltenyi Biotech) and 10 μM Y27632 dichloride (1254, R&D, Tocris) for 24h. The media was replaced every 2 days and the Y27632 dichloride supplement is no longer added. Once neural rosettes form, at day 17 of the differentiation, cells were passaged again as before, but instead they were plated in a 1:2 ratio. The final passage was performed at day 25, once neurons first begin to accumulate at the outside of rosettes. To dissociate into single cells, cultures were incubated with Accutase (A6964, Sigma) until the cells detached from the plate. Lifted cells were then passed through a 40 μm cell strainer to obtain single cells. These were seeded on laminin 111-coated plates (Biolamina) at 35,000 cells/cm^2^ (for immunofluorescence) and 80,000 cells/cm^2^ (for RT-qPCR, immunoblotting and SWATH-MS), and incubated with differentiation media supplemented with 10 μM Y27632 dichloride (1254, R&D, Tocris) for 24h. The media was then completed replaced with differentiation media supplemented with 20 ng/ml BDNF (450-02-100, Peprotech) and 20 ng/ml GDNF (450-10-100, Peprotech) and then 50% of the media was replenished every 2-3 days. Throughout the course of differentiation cultures were monitored and imaged routinely. At day 80 cells were harvested. The protocol for cortical neurogenesis was based on Shi et al. (Shi et al., 2012a, 2012b), but optimized for the iPSC model used.

#### Membrane integrity assays

Digitonin injury: Cells were treated with different concentrations of digitonin (0, 3, 5, 7, 10, 13, 15, 17, 20, 25 and 30 μg/ml) for 10 or 20 min, as indicated. To measure membrane integrity upon digitonin permeabilization, cells were then incubated with 2.5μg/ml Hoechst-33342 (H3570, Invitrogen) (Excitation 350, Emission 461) and 2 μg/ml propidium iodide (P4864, Sigma) (Excitation 535, Emission 617) and imaged using Celigo cytometer (Brooks Life Science Systems). Analysis was conducted using the Celigo Software Version 2.1 and biological triplicates were considered. Cell death percentage (i.e. the percentage of propidium iodide positive cells compared to the total number of cells) is a read-out of membrane permeability.

Laser injury: Cells were kept at 37°C in cell imaging media and were treated with 1 mg/ml FM1-43 (Invitrogen T3163) and 2.5 μg/ml Hoechst 33258 (Sigma Aldrich 861405) immediately before injury. A 1-2 μm circular region was irradiated using a 355 nm UV ablation laser (Rapp OptoElectronic) with the following settings: 2.6 % power, 200 Hz repetition rate, pulse energy > 60 μJ, pulse length < 4 ns. Images were acquired with the inverted microscope Eclipse Ti-E (Nikon) paired with the UltraVIEW VoX Spinning Disk (PerkinElmer) using the 63x objective. Cells were imaged for 350s (including at least 50s pre-injury). Control of hardware as well as intensity measurements were performed with PerkinElmers Volocity software. Plasma membrane integrity was measured by monitoring uptake of dye as a change in intracellular fluorescence during the course of imaging.

#### Proteomic screen

##### Rational

To identify the protein targets of CHIP two separate mass spectrometry screens were conducted on two CHIP cell models (iPSC and cortical neurons). In an attempt to account for the inherent variability of the cortical differentiation, the CHIP KO cortical neurons were compared to both control cell lines (the unedited parental line and a CRISPR/Cas9 clone expressing CHIP). For both screens biological and technical triplicates were included (of note, each biological replicate comprises of duplicate wells due to the minimum protein concentration required for FASP).

##### Sample preparation

iPSC and mature cortical neurons (day 80) were imaged prior to harvesting and lysed on the cell culture plate with 8M urea, 50 mM Hepes pH 8, 100 mM KCl, 1mM DTT and 0.5% (w/v) n-Dodecyl-β-D-maltoside (compatible with mass spectrometry). Protein lysates were centrifuged at 14000 g/20 min/8°C and supernatant was subsequently transferred to clean tubes. Protein concentration was assessed using the Micro BSA assay (ThermoFisher Scientific) according to the manufacturer’s instructions for the microplate procedure but optimized for minimal sample consumption. Samples were snap frozen in liquid nitrogen and stored at -80°C.

Cell lysates were processed using the filter-aided sample preparation (FASP) protocol, as described previously (Wiśniewski et al., 2009). Urea buffer (8 M Urea in 0.1 M Tris pH 8.5) was added to a 10 kD spin filter column (Sartorius Stedim Biotech, #VN01H02). Protein concentration was determined using the RC-DC assay (Bio-rad). Normalized sample (80-100 μg) was added into the spin filter column and was centrifuged at 14000 g for 15 min at 20 °C. Urea buffer was added again with 100 mM Tris (2-carboxyethyl) phosphine hydrochloride (C4706, Sigma) and mixed, for protein reduction. The column was incubated for 30 min on a shaking thermo-block at 37°C and 600 rpm and centrifugation for 15 min at 4,000 g and 20°C. Free sulfhydryl groups were alkylated using 300 mM iodoacetamide in urea buffer (I6125, Sigma). Protein alkylation took place in the dark at room temperature for 20 min followed by another centrifugation at 14000 g for 15 min. Ammonium bicarbonate (100 mM) was added to the column followed by centrifugation at 14000 g for 20 min. The column was placed in a new low-binding collecting tube (Axygen) and 50 mM ammonium bicarbonate was added along with trypsin diluted in trypsin buffer (Promega) at a 1:50 ratio (w/w) (trypsin:protein). The sample was mixed at 600 rpm for 1 h and then incubated overnight in a humidified chamber at 37°C. The peptides were eluted by centrifugation at 14,000 rom for 15 min at 20°C. Samples within the same experiment were prepared simultaneously.

Peptide concentration was measured using the Quantitative Colorimetric Peptide Assay (23275, Pierce, Thermo Scientific), following the manufacturer’s instructions. The absorbance of the reaction was measured using a TECAM spectrophotometer at 480 nm. Samples were desalted on C18 Micro spin columns (Harvard Apparatus) as described in (Bouchal et al., 2009) and dried in a SpeedVac at 35°C for 1 h or until no residual liquid is present. Samples were stored at -80°C.

##### SWATH-MS analysis

Desalted tryptic peptides were dissolved in 100 μl of 5 % acetonitrile (ACN), 0.05 % TFA and the peptide concentration was determined using a NanoDrop (Thermo Scientific, MA, USA). Samples were diluted in order to load approximately 2 ug of peptides a 3.7 μl injection to column. Peptide samples were separated by liquid chromatograph using Eksigent Ekspert nanoLC 400 (SCIEX) online connected to a TripleTOF 5600+ mass spectrometer (SCIEX). Samples were pre-concentrated on a cartridge trap column (300 μm i.d. × 5 mm) packed with C18 PepMap100 sorbent with 5 μm particle size (Thermo Scientific) using a mobile phase composed of 0.05 % trifluoroacetic acid (TFA) in 2 % ACN. Pre-concentrated peptides were separated on a capillary analytical column (75 μm i.d. × 500 mm) packed with C18 PepMap100 sorbent, 2 μm particle size (Thermo Fisher Scientific). Mobile phase A was composed of 0.1 % (v/v) formic acid (FA) in water while mobile phase B was composed of 0.1 % (v/v) FA in ACN. The analytical gradient started from 2% B, the proportion of mobile phase B increased linearly up to 40% B in 120 min, with a flow rate of 300 nl/min. The analytes were ionized by a nano-electrospray ion source, where the temperature and flow rate of the drying gas was set to 150°C and 12 psi. Voltage at the capillary emitter was 2.65 kV. Measurement of each biological replicate was repeated three times to give technical triplicates. SWATH-MS data were acquired in high sensitivity positive mode. Precursor range was set from 400 Da up to 1200 Da and it was divided to 67 precursor SWATH windows with 12Da width and 1 Da overlap. Accumulation per SWATH windows was set to 50.9 ms resulting in a 3.5 sec cycle time was 3.5 sec. Product ions were scanned in a range from 360 to 1360 Da. Rolling collision energy setting with 15 mV collision energy spread was used.

##### Data-dependent acquisition to generate an in-house spectral library

The spectral library sample was prepared by equimolar pooling of all samples. The spectral library was measured as described in (Faktor and Bouchal, 2016). Briefly, pooled spectral library samples were measured in information-dependent mode (IDA). Precursor range was set from 400 Da up to 1250 Da in MS mode and from 200 Da up to 1600 Da in MS/MS mode. Cycle time was set to 2.3 sec and during each cycle the top 20 most intensive precursor ions were fragmented. The precursor exclusion time was set to 12 sec. IDA data were searched against a Homo sapiens reference database (Uniprot+TrEmbl, 2016_02, 70005 entries) in ProteinPilot 4.5 (AB-SCIEX). The reference database contained decoy sequences of all proteins. Trypsin was set as a protease and carbamidomethyl was defined as a fixed modification. Tolerated mass error in MS1 and MS2 was set to predefined TripleTOF 5600 settings. No emphasis to a particular variable modification was given. The FDR calculation was performed by searching the MS/MS data against the decoy database. A total of 2087 proteins (FDR<1 %) and 8713 peptides (FDR<1 %) (see Table S2) were identified. The identified proteins and corresponding spectra from the resulting GROUP file were imported into SWATH™ Acquisition MicroApp 1.0 a plugin of PeakView 1.2.0.3. (AB-SCIEX) to generate a spectral library. A maximum of 4 peptides with no miscleavages, no post-translational modifications and peptide confidence above 99% per protein were imported into the spectral library. Each peptide was represented by a maximum of 6 transitions. The resulting spectral library contained 1424 proteins (FDR<1 %) and out of these 1353 proteins were quantitated. Transitions to build-up the spectral library are available in Table S2.

##### SWATH-MS data extraction and statistical analysis

SWATH data analysis was performed in a software platform intended for SWATH data analysis developed by AB-SCIEX. Quantitative data extraction was performed in SWATH™ Acquisition MicroApp 1.0 plugin running under PeakView 1.2.0.3 (AB-SCIEX) with the in-house spectral library prepared as described above. A maximum of 4 peptides per protein (if detectable) and a maximum of 6 product ions per peptide (if detectable) were used for protein quantitation. Quantitative SWATH-MS data were extracted using a method with ± 4 min extraction window around the expected retention time. The retention time window was determined based on retention time variability in the dataset. Product ion chromatograms of transitions were extracted ± 0.05 Da around the expected product ion mass. Peak areas for each transition were determined from extracted product ion chromatograms. Peak areas were further processed in MarkerView 1.2.1.1. (AB-SCIEX) on a peptide and protein level. The SRM like nature of SWATH-MS data enabled the determination of summed intensities per each protein by summing corresponding transition peak areas. Summed protein intensities were normalized on total ion current in MarkerView software. A t-test function in MarkerView software was used to perform pairwise t-testing of summed protein peak areas across compared conditions. The confidence of detected differences in a protein expression level between conditions (e.g. CHIP WT vs. CHIP KO) are described by p-value determined in MarkerView. The data were normalized by using of DESeq2 package (Love et al., 2014) implemented in R (R-Core-Team, 2018). Results were visualized in heatmaps by pheatmap (Kolde, 2019). Comparative analyses were assessed by Pearson’s correlation.

#### Pathway analysis

To identify GO terms (biological processes, molecular functions and cellular components) associated with the SWATH-MS datasets were analyzed by GOrilla (Eden et al., 2007, 2009), Database for Annotation, Visualization and Integrated Discovery (DAVID) (Huang et al., 2009a, 2009b) and Search Tool for the Retrieval of Interacting Genes/Proteins (STRING) (Szklarczyk et al., 2019) gene ontology analysis software. These bioinformatic tools use different statistical parameters and reference databases to identify associated GO terms.

To obtain GO terms using GOrilla (http://cbl-gorilla.cs.technion.ac.il/), a background list and target set is created from each SWATH-MS analysis (only gene symbols are included). The former includes all proteins identified, while the target set includes significantly changes proteins (having a fold change ≤0.67 and ≥1.5). The enrichment score for each GO term is calculated by a hypergeometric distribution: *(b/n)/(B/N)*, where *N* is the total number of genes, *B* is the number of genes associated with a specific GO term, *n* is the number of entries in the target set and *b* is the number of entries in the target set that are associated with a specific GO term. In addition to the enrichment score, the enrichment *p-value* is also generated for each GO term, which describes the probability of observing that *b* or more entries in the target set are associated with a given GO term, under the null assumption that all GO term occurrence in a ranked list are equiprobable (Eden et al., 2009). Also, an adjusted p-value using the FDR approach (*q-value*) is given and a threshold of 0.05 is considered significant. GO terms and their p-values derived from GOrilla were represented graphically using the REduce + VIsualize Gene Ontology (REVIGO) software (http://revigo.irb.hr/) (Supek et al., 2011). Graphs show GO terms identified from P/KO and WT/KO SWATH-MS analyses. When a particular GO term is found in both datasets, the average p-values was plotted. GO terms plotted are color-coded depending on their Log10(p-value).

Like GOrilla, DAVID (https://david.ncifcrf.gov/tools.jsp) searches for significantly associated GO terms using both a background list and a target set (proteins with ≤0.67 and ≥1.5 fold changes). However, DAVID differs from GOrilla because it allows exact fold changes for each protein to be included in the datasets (a feature that was used in our study). When looking at membrane proteins specifically, non-significant and significant membrane proteins were included in these datasets (using the same fold change cut-off criterium). For each GO term identified the following is given: (1) number of entries from the input list that are involved in a term (“count”), (2) gene-enrichment analysis presented as a Modified Fisher Exact p-value that ranges from 0 (corresponding to a perfect enrichment) to 1 (“p-value”), and (3) an adjusted p-value using Benjamini (“Benjamini”).

The Functional Enrichment Analysis tool of STRING was also used to identified significantly associated GO terms with the input dataset. Unlike previous platforms discussed, all proteins identified and their fold changes were taken into account when using STRING. *Homo sapiens* origin was selected. For each GO terms identified an Enrichment score and FDR are calculated (a threshold of 0.05 is considered). The software also uses hypergeometric testing and eleven functional pathway classification frameworks (Szklarczyk et al., 2019).

For pathway analysis and predictions of upstream regulators, the Ingenuity Pathway Analysis (IPA) software (Qiagen) was used. Predictive canonical pathway and upstream regulator analyses were performed using the following cut off: -log(*p*-value) > 1.3 (which corresponds to p-values of <0.05). Z-scores reflect the predicted activation state of the pathway or potential upstream regulator (a positive/negative z-score indicates activation/inhibition). At its basis, the observed protein expression changes (identified by MS) are associated to molecular networks and literature-derived regulation direction (activation/inhibition). Z-scores rather than p-values were included because they reflect directionality. This is a significant advance compared to other pathway analysis tools, which just focus on statistical enrichment in an overlap set of genes (Krämer et al., 2014). Of note, this software was also used to isolate membrane proteins from all the SWATH-MS data.

### Quantification and statistical analysis

Quantitative reverse transcription PCR (qRT-PCR) was performed in biological duplicates and technical triplicates. Relative levels of transcript expression were quantified by the comparative CT method with normalization to *TBP* levels.

Quantification of SWATH-MS data and the statistical parameters used are described in the section “SWATH-MS analysis” under Method details. Biological triplicates (each are a pool of duplicates) and technical triplicates were included.

Membrane integrity was assessed by quantifying the intracellular fluorescence of proprium iodide or FM4-64 dye (added extracellularly) upon injury by digitonin or UV laser, respectively. The number of cells injured by digitonin were represented as a percentage of all cells quantified. Mean ± SD of biological triplicates were plotted and were representative of three independent experiments. Data passed the D’Agostino-Pearson normality test (alpha=0.05) and was analyzed by two-way ANOVA with Holm-Sidak’s multiple comparison post-hoc test. With regards to cells injured by UV laser, the intracellular fluorescence intensity (F) changes over time were analyzed by calculating the mean of the normalized intensity values (FΔ/F0) as a function of time for each cell body (n ≥ 16 cells), yielding area under the curve (AUC) values. Data passed the D’Agostino-Pearson normality test (alpha=0.05) and a *Student’s t-test* was performed on the AUC values of both cell lines (CHIP KO and WT) under the null hypothesis that changes in fluorescence intensities over time are the same for different groups. Mean ± SEM was plotted. Quantification of fluorescence intensity was performed using the Volocity software.

Normality tests and statistical analysis were performed using GraphPad Prism 8 (GraphPad software, Inc.). Statistical tests used and p values are detailed in figure legends (Figures 1I, 2F, 4H, 4I, and 4K), with significance established at p value < 0.05. Graphs and illustrations were performed with GraphPad Prism and Illustrator software (Adobe).

## Supplemental information

Document S1. Figures S1–S7 and Tables S1–S10

Data S1. ▪▪▪

Data S2. ▪▪▪

Data S3. ▪▪▪

Data S4. ▪▪▪
