# CHIP-dependent regulation of the actin cytoskeleton is linked to neuronal cell membrane integrity

**DOI:** 10.1016/j.isci.2022.103921

**Published:** 2022-02-23

**Authors:** Catarina Dias, Erisa Nita, Jakub Faktor, Ailish C. Tynan, Lenka Hernychova, Borivoj Vojtesek, Jesper Nylandsted, Ted R. Hupp, Tilo Kunath, Kathryn L. Ball

## Main text

(iScience *24*, 102878, August 20, 2021.)

Stylistic changes to the manuscript were made for clarity (and involved the addition of a single vertical black line to Figure 1K). This indicates that although the two samples were run on the same gel, they were not in adjacent lanes (they were originally separated by a CHIP heterozygous cell sample). In addition, the legend for Figure 2I is modified, showing that the ANXA2 data came from re-probing the membrane shown in Figure 1K (in this case including the CHIP heterozygous cell sample). As a result, some of the β-actin and CHIP controls are common to both Figures 1K and 2I.

In the supplemental information, white lines were added to Figure S1. The PCR data were taken from a larger screen, and the samples may not have been in adjacent lanes on the agarose gel. Further, Figures S7A and S7B have been consolidated into a single figure (Figure S7A) containing all the original data. The figure legends have been edited to reflect this change.

All the authors agree that the changes have no impact on the outcome or conclusions of the study, as no data has been removed or added post publication of the original manuscript. The authors apologize for any confusion caused to the readers.

**Figure:**
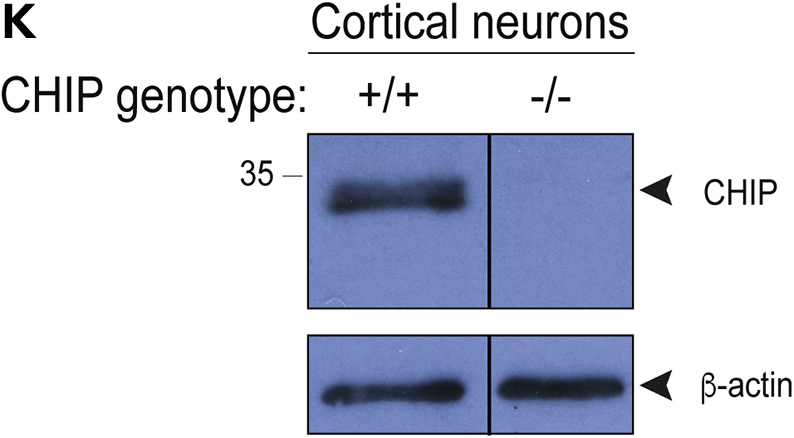
Figure 1K. Isogenic CHIP patient-derived iPSC and cortical models (corrected)

**Figure:**
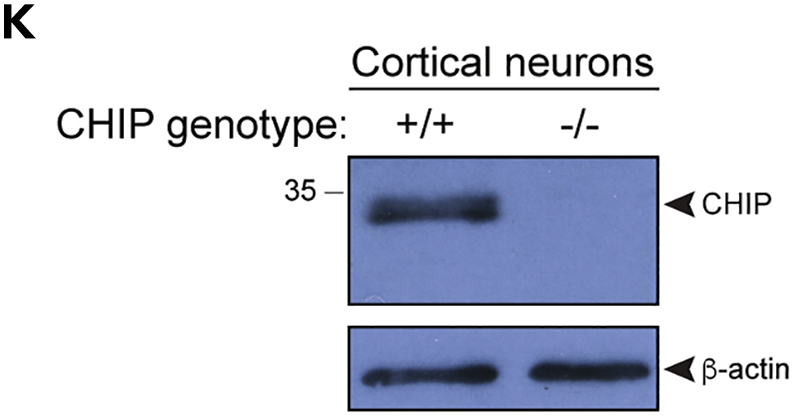
Figure 1K. Isogenic CHIP patient-derived iPSC and cortical models (original)

**Figure:**
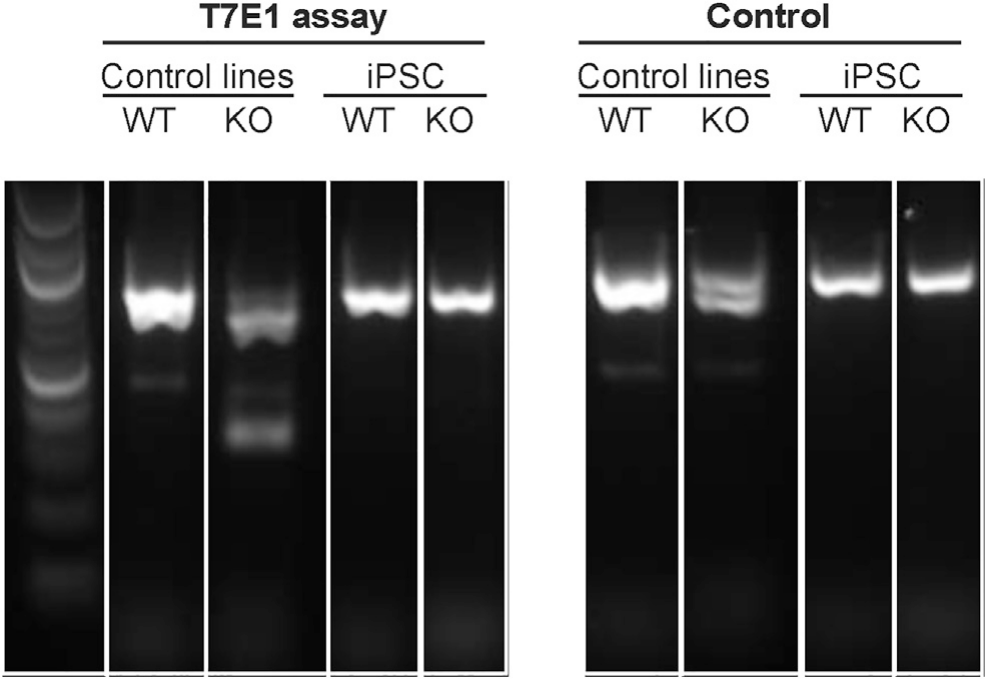
Figure S1 (corrected)

**Figure:**
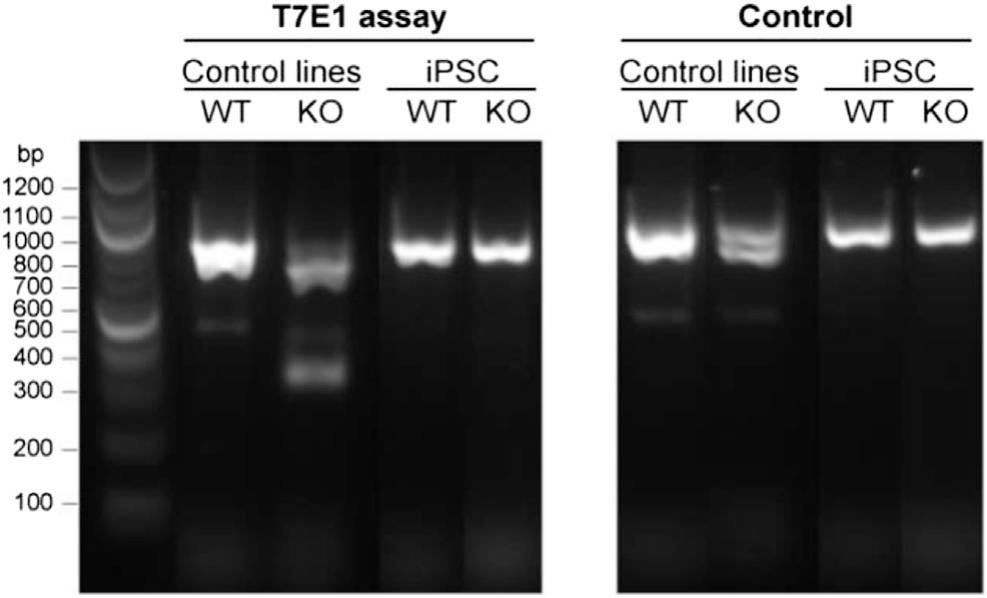
Figure S1 (original)

**Figure:**
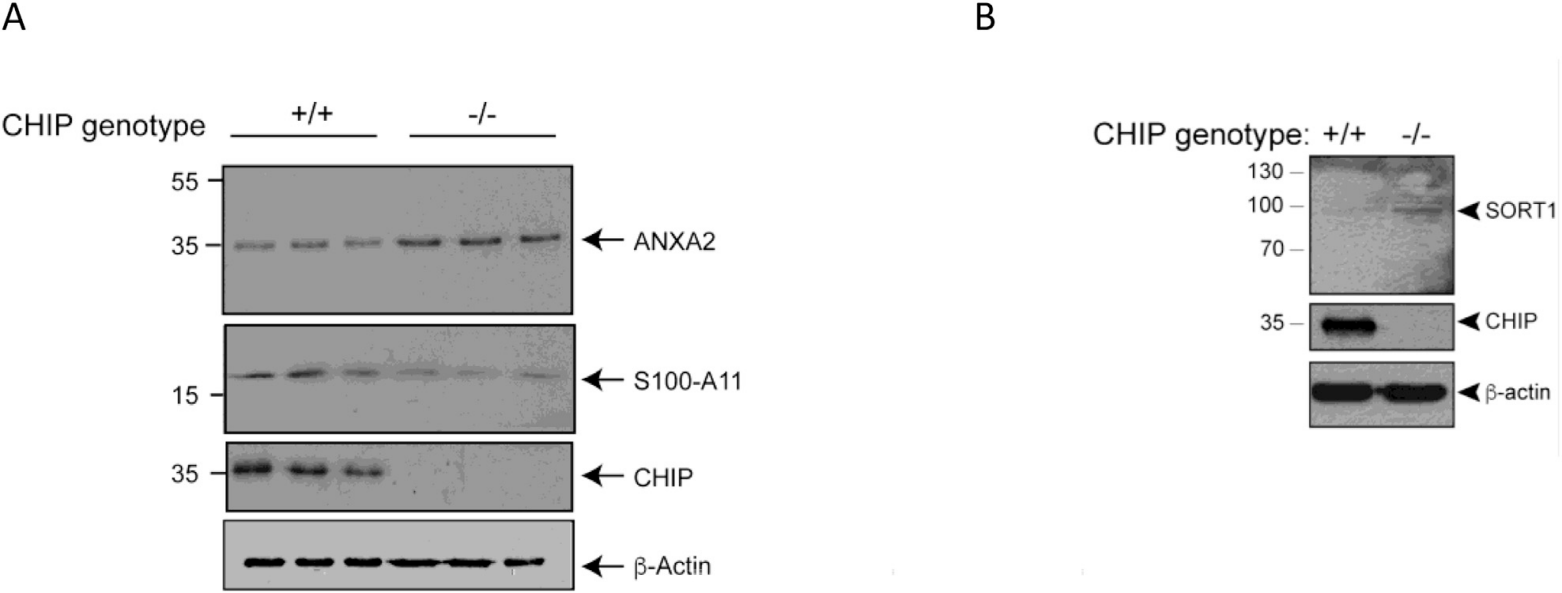
Figure S7 (corrected)

**Figure:**
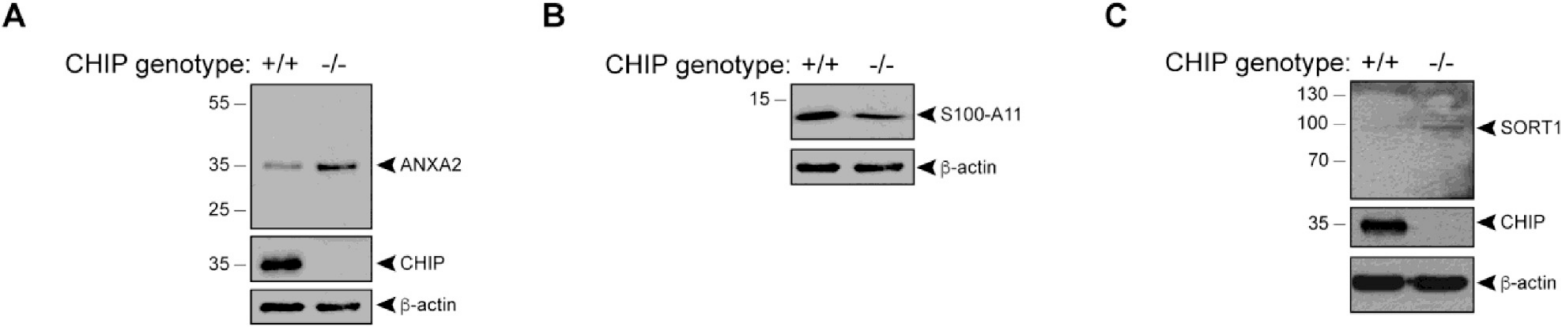
Figure S7 (original)

