# Supplementary material for: CHIP-dependent regulation of the actin cytoskeleton is linked to neuronal cell membrane integrity
Source: iScience. 2021 Jul 17;24(8):102878. doi: 10.1016/j.isci.2021.102878 (PMC8350547; doi:10.1016/j.isci.2021.102878)
Supplement: Document S1. Figures S1–S7 and Tables S1–S10 [file mmc1.pdf]

iScience, Volume *24*

Supplemental information

CHIP-dependent regulation of the actin cytoskeleton is linked to neuronal cell membrane integrity

Catarina Dias, Erisa Nita, Jakub Faktor, Ailish C. Tynan, Lenka Hernychova, Borivoj Vojtesek, Jesper Nylandsted, Ted R. Hupp, Tilo Kunath, and Kathryn L. Ball

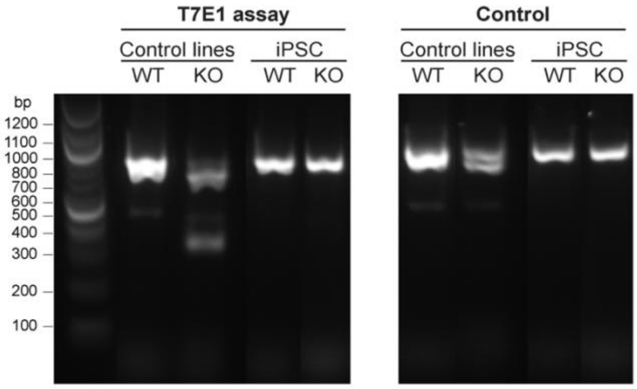

Figure S1. Homologous indel in the CHIP KO iPSC line. Related to Figure 1.

The homozygosity of the PCR-amplified *STUB1* locus of the CRISPR/Cas9-derived iPSC clones was assessed using the T7 endonuclease assay. Previously characterised CHIP WT and KO SH-SY5Y were included as negative and positive controls. The enzyme recognises and cleaves non-homologous dsDNA, detecting indels. No cleavage event was detected in the CHIP WT and KO iPSC lines

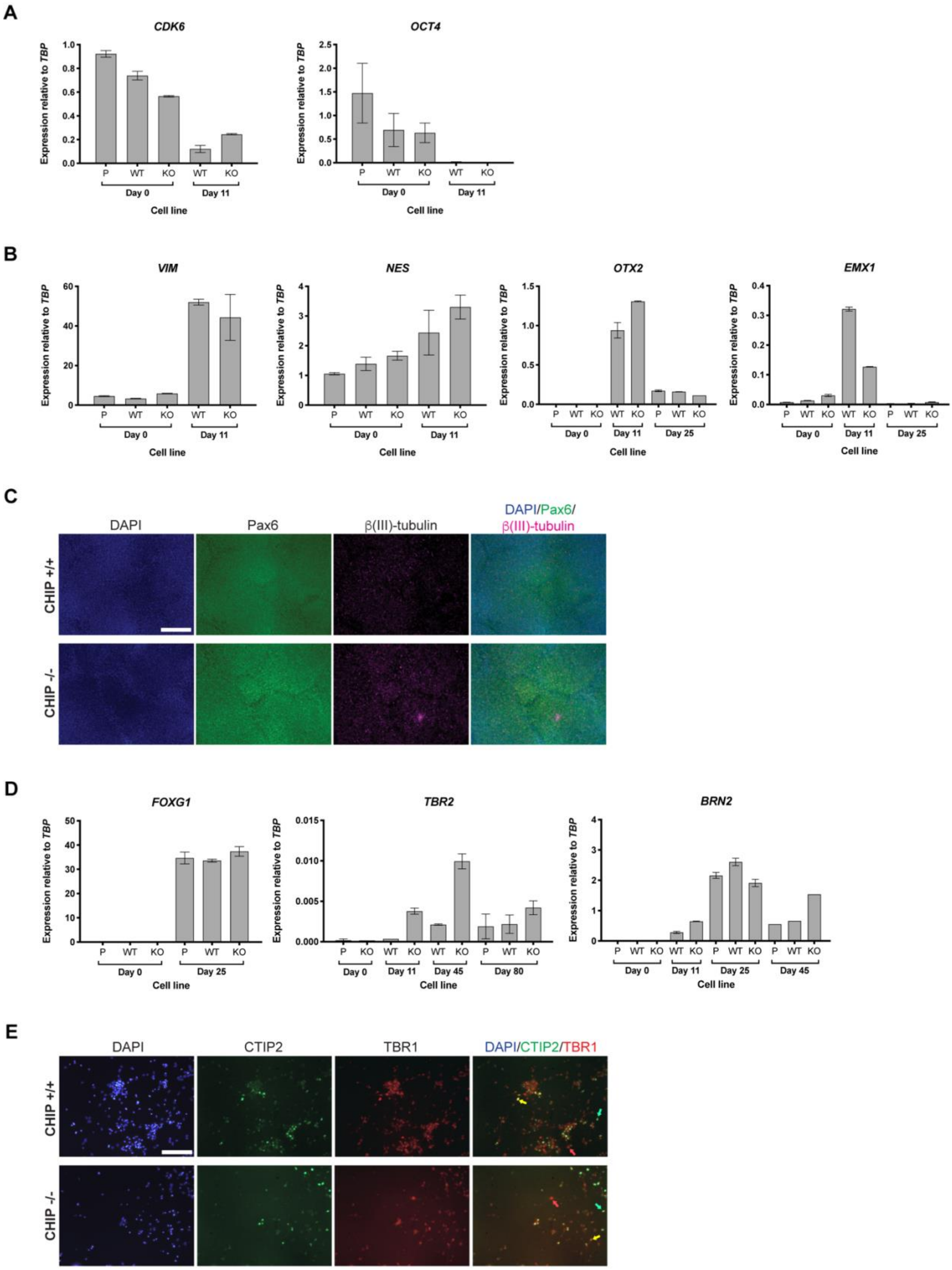
A
CDK6
OCT4
Expression relative to TBP
P WT KO WT KO
Day 0
Day 11
Cell line
B
VIM
NES
OTX2
EMX1
Day 25
C
DAPI
Pax6
β(III)-tubulin
DAPI/Pax6/β(III)-tubulin
CHIP +/+
CHIP -/-
D
FOXG1
TBR2
BRN2
Day 45
Day 80
E
CTIP2
TBR1
DAPI/CTIP2/TBR1

Figure S2. Characterisation of the isogenic CHIP neuronal model. Related to Figure 1.

A) Expression of pluripotency markers (*CDK6* and *OCT4*) assessed by qRT-PCR (normalised by TATA binding box protein (*TBP*) gene expression) in our isogenic CHIP model throughout the differentiation process. Bars represent averages and error bars represent standard deviations of three technical replicates. Results are representative of two independent cortical differentiations. B) Expression of markers of cortical stem and progenitor cells (*VIM, NES, OTX2* and *EMX1*) assessed by qRT-PCR (normalised by TATA binding box protein (*TBP*) gene expression) in our isogenic CHIP model throughout the differentiation process. Bars represent averages and error bars represent standard deviations of three technical replicates. Results are representative of two independent cortical differentiations. C) Representative images of the expression of the neuroectodermal marker, PAX6, in mature CHIP-expressing and KO cortical neurons (at day 11 of the differentiation). Scale bar, 200 µm. D) Expression of markers of secondary progenitor and stem cells (*FOXG1* and *TBR2*) assessed by qRT-PCR (normalised by TATA binding box protein (*TBP*) gene expression) in our isogenic CHIP model throughout the differentiation process. Bars represent averages and error bars represent standard deviations of three technical replicates. Results are representative of two independent cortical differentiations. E) Representative images of the expression of deep-layer makers, CTIP2 and TBR1, in mature CHIP-expressing and KO cortical neurons (at day 80 of the differentiation). Scale bar, 100 µm.

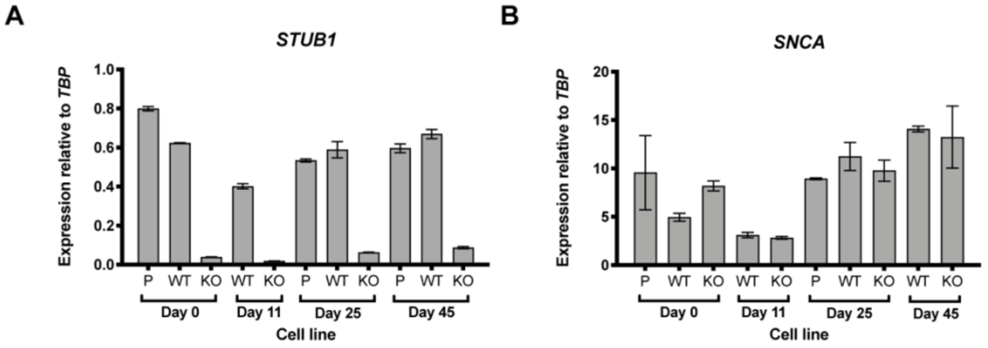

Figure S3. Expression of *STUB1* and *SNCA* throughout the course of differentiation. Related to Figure 1.

The expression of *STUB1* (**A**) and *SNCA* (**B**) throughout the course of differentiation assessed by qRT-PCR (normalised by TATA binding box protein (*TBP*) gene expression). As expected, the later increases and neurons mature, while *STUB1* expression remains relatively unchanged. Bars represent averages and error bars represent standard deviations of three technical replicates. Results are representative of two independent cortical differentiations.

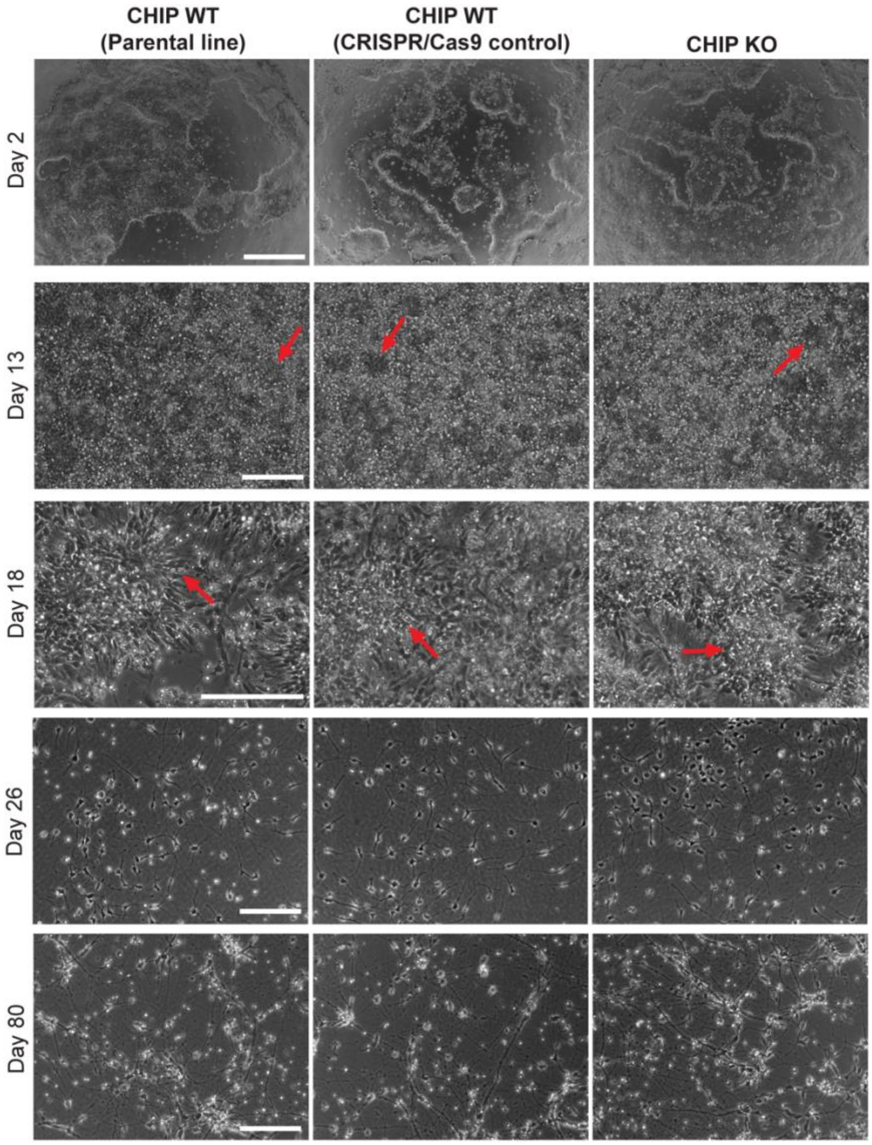

Figure S4. Overview of the morphology of neuronal cultures throughout the differentiation. Related to Figure 1.

Phase contrast images of the CHIP-expressing lines (parental and CRISPR/Cas9 control) and CHIP KO line throughout the course of differentiation. Cell shift from the typical morphology of iPSC (observed at day 2), to that of neuronal progenitors (forming rosettes that are denoted by the arrows and more visible in the zoomed-out inlet) and developing neurons (visible from day 26). At all stages of the differentiation the morphology and confluency across cell lines is highly similar. Scale bars, 200 µm (images taken at days 13, 18, 26 and 80) and 80 µm (day 2).

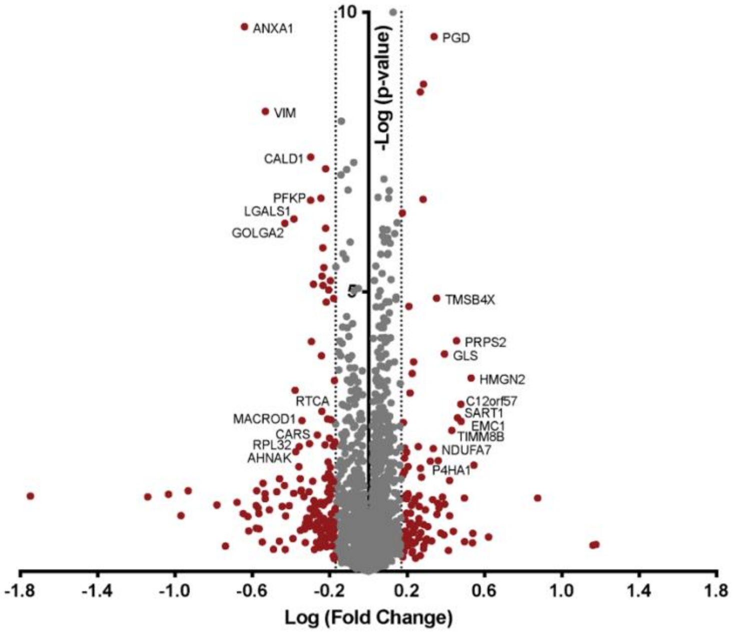

Figure S5. Proteomic analysis of the isogenic CHIP iPSC model. Related to Figure 2.

SWATH-MS analyses of the CHIP iPSC model (WT/KO). Cut off criteria (≤0.67 and ≥1.5 fold change) are represented by the dashed lines. Proteins that do not meet this fold change criteria are colour-coded in grey and does that do are in red. Over-represented proteins in the CHIP KO sample compared to WT have negative log (fold change), while under-represented proteins have positive values. The top 11 most under- and over-represented proteins that were significantly changed ($P < 0.05$) are annotated.

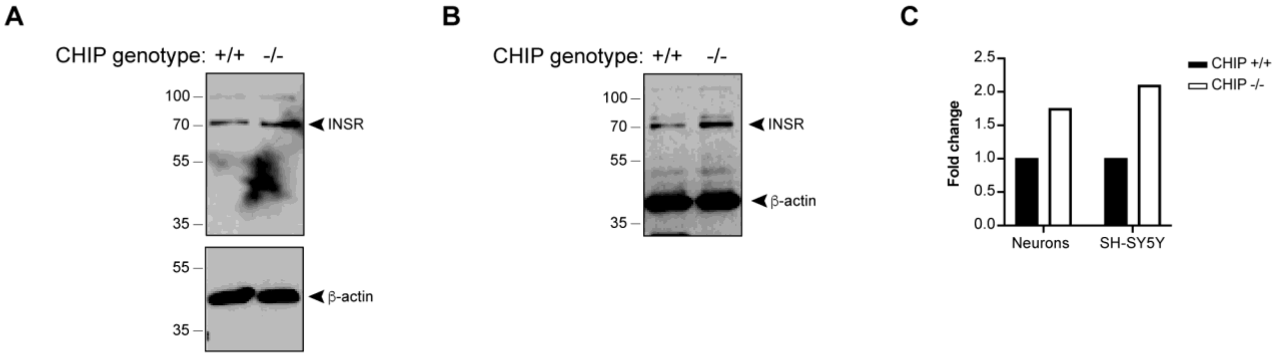

Figure S6. Proteomic changes in the CHIP SH-SY5Y model. Related to Figure 4.
Steady state levels of SORT1 (A), ANXA2 (B) and S100A11 (C) in the isogenic CHIP SH-SY5Y model detected by SDS-PAGE/immunoblot.

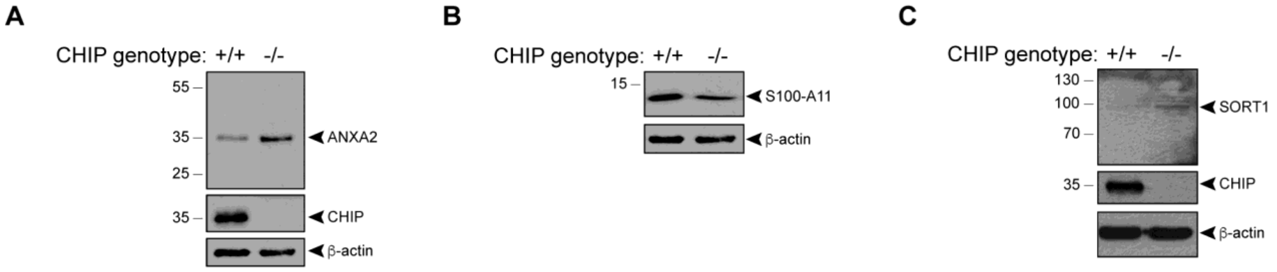

Figure S7. Changes in the steady state levels of INSR in the CHIP neuronal and SH-SY5Y models. Related to Figure 4. The steady state levels of INSR in both the isogenic CHIP neuronal model (A) and SH-SY5Y model (B) were also detected by SDS-PAGE/immunoblot and quantified in a histogram where relative density was normalised to the CHIP WT cell line (C).

Table S1. The most under- and over-represented proteins in the SWATH-MS analysis comparing parental cortical neurons (genetically unedited) to CHIP KO cortical neurons (i.e. P/KO). Related to Figure 2.

	Protein	p-value	Fold change	-Log (p-value)	Log (Fold change)
Under-represented proteins	Statherin	7.00E-02	0.080	1.155	-1.097
	Glial fibrillary acidic protein	2.38E-03	0.091	2.623	-1.042
	Annexin A2	1.61E-06	0.100	5.794	-0.999
	Interferon-induced transmembrane protein 2	6.72E-10	0.103	9.173	-0.988
	Galectin-3	4.56E-06	0.108	5.341	-0.968
	Caldesmon	1.30E-04	0.168	3.886	-0.775
	Protein S100-A11	6.19E-05	0.184	4.208	-0.734
	Prelamin-A/C	3.48E-06	0.238	5.459	-0.623
	HLA class I histocompatibility antigen, B-78 alpha chain	2.46E-05	0.264	4.609	-0.579
	Parkinson disease 7 domain-containing protein 1 (Fragment)	6.61E-02	0.271	1.180	-0.567
	Annexin A1	2.09E-07	0.274	6.680	-0.563
	Guanine nucleotide-binding protein G(I)/G(S)/G(O) subunit gamma-12	2.23E-07	0.274	6.652	-0.563
Over-represented proteins	Disks large homolog 4	0.04393	12.044	1.357	1.081
	E3 ubiquitin-protein ligase CHIP	7.98E-03	11.699	2.098	1.068
	Corticoliberin	6.60E-04	5.913	3.180	0.772
	STE20/SPS1-related proline-alanine-rich protein kinase	1.38E-01	5.056	0.859	0.704
	Propionyl-CoA carboxylase beta chain, mitochondrial	4.09E-01	4.248	0.389	0.628
	Sclerostin domain containing protein 1	7.46E-07	4.213	6.127	0.625
	Keratin, type II cuticular Hb2	7.32E-02	3.316	1.135	0.521
	Poliovirus receptor	1.44E-01	2.349	0.843	0.371
	Caytaxin	8.20E-02	2.344	1.086	0.370
	Metallophosphoesterase MPPED2	7.08E-09	2.337	8.150	0.369
	Amine oxidase [flavincontaining] A	5.61E-06	2.250	5.251	0.352
	Synaptotagmin-5	0.0262	2.247	1.582	0.352
	Caseinolytic peptidase B protein homolog	2.86E-03	2.243	2.544	0.351
	F-box only protein 2	4.62E-05	2.217	4.336	0.346
	GTP-binding protein 1	1.73E-01	2.163	0.761	0.335
	U2 small nuclear ribonucleoprotein B''	2.50E-01	2.118	0.603	0.326
	Serine/threonine-protein phosphatase 6 regulatory subunit 2 (Fragment)	1.87E-01	2.048	0.728	0.311
	WD repeat-containing protein 44	2.43E-16	2.041	15.614	0.310

Table S2. The most under- and over-represented proteins in the SWATH-MS analysis comparing CHIP-expressing cortical neurons (derived from a CRISPR/Cas9 control clone) to CHIP KO cortical neurons (i.e. WT/KO). Related to Figure 2.

	**Protein**	**p-value**	**Fold change**	**-Log (p-value)**	**Log (Fold change)**
**Under-represented proteins**	Interferon-induced transmembrane protein 2	5.84E-10	0.098	9.234	-1.007
	Annexin A2	1.66E-06	0.107	5.779	-0.969
	Glial fibrillary acidic protein	2.78E-03	0.109	2.556	-0.961
	Caldesmon	5.89E-05	0.116	4.230	-0.936
	Galectin-3	5.51E-06	0.120	5.259	-0.920
	Transgelin-2	4.49E-06	0.148	5.348	-0.831
	Annexin A1	3.25E-08	0.171	7.489	-0.768
	HLA class I histocompatibility antigen, B-78 alpha chain	6.51E-06	0.171	5.186	-0.767
	PDZ and LIM domain protein 5	2.27E-07	0.181	6.643	-0.743
	Clusterin	8.83E-10	0.190	9.054	-0.722
	Protein S100-A11	8.47E-05	0.206	4.072	-0.686
**Over-represented proteins**	Corticoliberin	2.98E-05	5.385	4.526	0.731
	E3 ubiquitin-protein ligase CHIP	1.37E-01	4.444	0.862	0.648
	Sclerostin domaincontaining protein 1	1.51E-05	3.594	4.821	0.556
	F-box only protein 2	4.37E-06	2.667	5.360	0.426
	Glutaredoxin-related protein 5, mitochondrial	1.94E-01	2.452	0.712	0.390
	Semaphorin-3C	1.87E-09	2.375	8.729	0.376
	AP-3 complex subunit mu-2	1.25E-01	2.366	0.905	0.374
	Synaptosomal-associated protein 29	8.76E-02	2.254	1.057	0.353
	RNA-binding protein Musashi homolog 1	1.36E-01	2.247	0.865	0.352
	Testican-1	2.18E-10	2.241	9.661	0.350
	Metallophosphoesterase MPPED2	6.30E-07	2.203	6.200	0.343
	Synaptotagmin-5	8.34E-05	2.187	4.079	0.340
	Histone H2A type 2-A	6.30E-04	2.185	3.201	0.339
	Caytaxin	1.04E-01	2.179	0.982	0.338
	Neurofilament light polypeptide	6.54E-06	2.154	5.184	0.333
	Brain-derived neurotrophic factor	9.55E-11	2.142	10.020	0.331

Table S3. The most under- and over-represented proteins in the SWATH-MS analysis comparing CHIP-expressing cortical neurons (the parental line and the CRISPR/Cas9 control line) (i.e. P/WT). Related to Figure 2.

	Protein	p-value	Fold change	-Log (p-value)	Log (Fold change)
Under-represented proteins	Statherin	0.02645	0.086	1.578	-1.067
	Semenogelin-1	5.02E-02	0.271	1.299	-0.568
	Histone H2A type 2-A	6.77E-06	0.271	5.169	-0.567
	Filaggrin-2	1.76E-02	0.320	1.755	-0.495
	Coronin	1.24E-01	0.323	0.906	-0.491
	NADH dehydrogenase [ubiquinone] 1 beta subcomplex subunit 6	9.32E-03	0.391	2.031	-0.408
	Dermcidin	2.01E-03	0.402	2.697	-0.395
	Prolactin-inducible protein	5.75E-02	0.426	1.240	-0.371
	Sarcolemmal membrane associated protein	3.15E-03	0.429	2.502	-0.368
	Brain-derived neurotrophic factor	4.11E-12	0.444	11.386	-0.352
	RNA-binding protein Musashi homolog 1	1.34E-01	0.445	0.872	-0.351
	Protein BORCS7-ASMT	1.54E-01	0.469	0.814	-0.329
	Apolipoprotein C-III	1.91E-06	0.475	5.719	-0.323
	Annexin A4	5.73E-09	0.488	8.242	-0.311
	Junctional adhesion molecule C	1.63E-02	0.494	1.789	-0.307
Over-represented proteins	GTP-binding protein 1	1.90E-02	8.272	1.721	0.918
	STE20/SPS1-related proline-alanine-rich protein kinase	1.27E-01	5.771	0.895	0.761
	Pirin	1.12E-07	3.553	6.952	0.551
	Lysosome-associated membrane glycoprotein 5	2.18E-01	3.463	0.662	0.539
	Amyloid-like protein 2	4.30E-04	3.102	3.367	0.492
	WD repeat-containing protein 18 (Fragment)	2.42E-02	2.682	1.617	0.428
	E3 ubiquitin-protein ligase CHIP	9.94E-02	2.636	1.003	0.421
	Symplekin	1.32E-01	2.541	0.880	0.405
	Fermitin family homolog 2 (Fragment)	1.64E-01	2.538	0.784	0.405
	Serine/threonine-protein phosphatase 6 regulatory subunit 2 (Fragment)	9.87E-02	2.507	1.006	0.399
	U2 small nuclear ribonucleoprotein B''	1.95E-01	2.468	0.709	0.392
	Peptidyl-prolyl cis-trans isomerase NIMAinteracting 4	1.06E-05	2.436	4.974	0.387
	FYVE, RhoGEF and PH domain-containing protein 4	4.27E-02	2.433	1.369	0.386
	Allograft inflammatory factor 1-like	9.11E-02	2.430	1.040	0.386
	Glutathione S-transferase omega-1	1.73E-02	2.373	1.762	0.375
	Histone H3.1	1.42E-03	2.265	2.848	0.355
	Transcription elongation factor SPT5	3.33E-01	2.262	0.478	0.355
	mRNA export factor	6.43E-02	2.254	1.192	0.353
	Vesicle transport through interaction with t-SNAREs homolog 1A	8.32E-03	2.253	2.080	0.353
	Aminopeptidase B	3.44E-03	2.225	2.463	0.347
	RNA-binding protein 3	1.04E-03	2.199	2.983	0.342
	1-acyl-sn-glycerol-3-phosphate acyltransferase alpha	4.90E-04	2.167	3.310	0.336

Table S4. Proteins potentially influenced by CHIP expression in cortical neurons. Related to Figure 2.

Significantly changed proteins (with fold changes of ≤0.5 and ≥2) in the SWATH-MS analyses comparing CHIP-expressing cortical neurons (the parental line and the CRISPR/Cas9 control line) to CHIP KO cortical neurons (i.e. P/KO and WT/KO) that show the opposite trend or no change in the SWATH-MS analysis comparing CHIP-expressing cortical neurons (the parental line and the CRISPR/Cas9 control line) (i.e. P/WT).

		P/KO		WT/KO		P/WT	
**Proteins**		**Fold change**	**p-value**	**Fold change**	**p-value**	**Fold change**	**p-value**
IFITM2	Interferon-induced transmembrane protein 2	0.103	6.72E-10	0.098	5.84E-10	-	-
ANXA2	Annexin A2	0.100	1.61E-06	0.107	1.66E-06	0.964	8.61E-01
GFAP	Glial fibrillary acidic protein	0.091	2.38E-03	0.109	2.78E-03	-	-
CALD1	Caldesmon	0.168	1.30E-04	0.116	5.89E-05	1.704	4.92E-02
LGALS3	Galectin-3	0.108	4.56E-06	0.120	5.51E-06	0.664	1.97E-01
TAGLN2	Transgelin-2	0.275	8.41E-06	0.148	4.49E-06	1.845	1.37E-05
ANXA1	Annexin A1	0.274	2.09E-07	0.171	3.25E-08	1.603	1.80E-04
HLA-B	HLA class I histocompatibility antigen, B-78 alpha chain	0.264	2.46E-05	0.171	6.51E-06	1.544	7.10E-02
CLU	Clusterin	0.291	6.09E-09	0.190	8.83E-10	1.535	2.82E-07
S100A11	Protein S100-A11	0.184	6.19E-05	0.206	8.47E-05	0.800	2.59E-01
GNG12	Guanine nucleotide-binding protein G(I)/G(S)/G(O) subunit gamma-12	0.274	2.23E-07	0.207	6.63E-08	1.321	1.82E-01
KRT6B	Keratin type II cytoskeletal 6B	0.342	9.24E-02	0.213	4.21E-02	1.603	2.75E-01
LMNA	Prelamin-A/C	0.238	3.48E-06	0.257	6.47E-06	0.928	7.51 E-01
CECR5	Cat eye syndrome critical region protein 5	0.452	1.63E-01	0.266	7.03E-02	1.701	1.77E-01
TAGLN	Transgelin	0.337	4.18E-03	0.311	2.27E-03	1.845	1.37E-05
SERPINH1	Serpin H1	0.343	6.52E-09	0.316	5.00E-09	1.086	7.29E-02
GSN	Gelsolin	0.347	5.25E-08	0.320	5.53E-08	1.093	2.59E-01
FLNA	Filamin-A	0.388	1.35E-06	0.323	3.42E-07	1.203	1.40E-04
HTRA1	Serine protease HTRA 1	0.371	1.43E-06	0.326	1.03E-06	1.140	2.72E-01
AHNAK	Neuroblast differentiation-associated protein AHNAK	0.349	1.72E-02	0.328	1.24E-02	-	-
ZFP91-CNTF	HCG2042749, isoform CRA_d	0.302	5.47E-02	0.348	7.26E-02	0.871	6.81E-01
LGALS1	Galectin-1	0.320	2.30E-04	0.363	5.10E-03	-	-
KRT6A	Keratin type II cytoskeletal 6A	0.356	4.43E-02	0.374	6.04E-02	0.951	9.11E-01
ANXA6	Annexin A6	0.441	1.70E-04	0.382	6.20E-05	1.156	8.22E-06
TUBB6	Tubulin beta-6 chain	0.487	7.60E-14	0.395	2.41E-14	1.233	5.S0E-04
ANXA5	Annexin AS	0.454	3.33E-07	0.406	1.02E-07	1.121	1.31 E-02
MOK	Midkine	0.310	1.42E-09	0.426	2.57E-08	0.728	2.49E-03
PTBP1	Polypyrimidine tract binding protein 1, isoform CRAb	0.471	2.92E-10	0.427	2.94E-09	1.106	3.27E-01
PDDC1	Parkinson disease 7 domain-containing protein 1	0.271	6.61E-02	0.436	2.23E-01	0.622	5.56E-01
PALLD	Palladin	0.500	6.30E-04	0.457	2.40E-04	1.094	5.24E-01
ACTN1	Alpha-actinin-1	0.380	2.30E-04	0.461	9.60E-04	1.026	6.54E-01
USP9X	Probable ubiquitin carboxyl-terminal hydrolase FAF-X	0.439	2.55E-06	0.487	4.80E-06	0.902	6.52E-01
TBR1	T-box brain protein 1	0.400	5.S0E-03	0.498	1.0SE-02	0.803	6.0SE-01
ATCAY	Cavtaxin	2.344	8.20E-02	2.179	1.04E-01	1.077	8.43E-01
SYT5	Synaptotagmin-5	2.247	2.62E-02	2.187	8.34E-05	1.028	9.00E-01
MPPED2	Metallophosphoesterase MPPED2	2.337	7.08E-09	2.203	6.30E-07	1.062	3.16E-01
FBXO2	F-box only protein 2	2.217	4.62E-05	2.667	4.37E-06	0.832	4.66E-03
SOSTDC1	Sclerostin domain-containing protein 1	4.213	7.46E-07	3.594	1.51 E-05	1.173	1.85E-01
CRH	Corticoliberin	5.913	6.60E-04	5.385	2.98E-05	1.099	6.99E-01

Table S5. GO terms related to CHIP loss-of-function. Related to Figure 3.

Biological process, molecular function and cellular component GO terms identified in the P/KO and WT/KO SWATH-MS datasets and excluded from P/WT (using GOrilla, DAVID and STRING). The number of associated genes ("count") and an association index (probability (p-value, FDR q-value and/or Benjamini) and/or score) for each GO term is included.

Biological Process					Molecular Function					Cellular Component				
GO term			P/KO	WT/KO	GO term			P/KO	WT/KO	GO term			P/KO	WT/KO
Cell-cell adhesion	GOrilla	Count	16	22	Actin binding	GOrilla	Count	26	26	Plasma membrane	GOrilla	Count	79	91
		p-value	9.96 E-04	1.32 E-05			p-value	8.63 E-06	3.32 E-04			p-value	1.61 E-04	8.36 E-05
		FDR q-value	4.26 E-01	3.95 E-02			FDR q-value	1.94 E-02	1.85 E-01			FDR q-value	2.02 E-02	2.63 E-02
		Enrich-ment	2.28	2.54			Enrich-ment	2.41	1.96			Enrich-ment	3.71	3.10
	DAVID	Count	16	17		DAVID	Count	17	19		DAVID	Count	66	88
		p-value	1.70 E-06	8.10 E-06			p-value	4.4 E-07	4.3 E-07			p-value	7.90 E-03	2.90 E-04
		Benja-mini	2.20 E-03	6.80 E-03			Benja-mini	6.2 E-05	5.4 E-05			Benja-mini	9.60 E-02	7.40 E-03
Cytoskeleton organization	DAVID	Count	9	9	Calcium ion binding	DAVID	Count	20	18	Neuron projection	DAVID	Count	10	12
		p-value	9.60 E-04	2.3 E-01			p-value	1.7 E-03	6.0 E-02			p-value	2.30 E-03	9.60 E-04
		Benja-mini	2.3 E-01	4.4 E-01			Benja-mini	8.5 E-02	6.6 E-01			Benja-mini	3.90 E-02	1.90 E-02
Regulation of cell migration	DAVID	Count	5	7	S100 protein binding	DAVID	Count	6	6	Microtubule	DAVID	Count	10	11
		p-value	1.4 E-02	1.2 E-03			p-value	7.8 E-04	1.3 E-02			p-value	1.30 E-02	2.00 E-02
		Benja-mini	6.9 E-01	2.5 E-01			Benja-mini	4.6 E-02	3.2 E-02			Benja-mini	1.40 E-01	1.60 E-01
Membrane fusion	DAVID	Count	4	4		STRING	Count	7	7	Synaptic vesicle	DAVID	Count	11	9
		p-value	1.8 E-02	3.3 E-02			Enrich-ment	361.74	391.31			p-value	1.50 E-07	7.40 E-04
		Benja-mini	6.6 E-01	7.9 E-01			FDR q-value	4.2 E-04	1.6 E-03			Benja-mini	9.90 E-06	2.60 E-03
Regulation of exocytosis	DAVID	Count	3	4	Microtubule binding	DAVID	Count	9	8	Ruffle	DAVID	Count	7	9
		p-value	4.2 E-02	8.0 E-03			p-value	4.7 E-03	4.6 E-02			p-value	7.40 E-04	6.30 E-05
		Benja-mini	8.2 E-01	4.9 E-01			Benja-mini	2.0 E-01	5.8 E-01			Benja-mini	1.40 E-02	2.50 E-03
Neurotransmitter secretion	DAVID	Count	5	4	Cytoskeletal protein binding	DAVID	Count	4	7	Neurofibrillary tangle	DAVID	Count	5	6
		p-value	3.8 E-03	4.8 E-02			p-value	2.2 E-02	1.0 E-04			p-value	4.20 E-03	1.40 E-03
		Benja-mini	4.0 E-01	8.2 E-01			Benja-mini	4.2 E-01	7.1 E-03			Benja-mini	5.40 E-02	2.40 E-02
Axon extension	DAVID	Count	4	3	Structural molecule activity	STRING	Count	166	165	Synaptic vesicle membrane	DAVID	Count	5	6
		p-value	3.2 E-03	5.6 E-02			Enrich-ment	7.32 E-01	5.83 E-01			p-value	4.20 E-03	1.40 E-03
		Benja-mini	3.8 E-01	8.5 E-01			FDR q-value	5.39 E-11	2.80 E-04			Benja-mini	5.40 E-02	2.40 E-02
Protein targeting to membrane	STRING	Count	85	85	Calcium-dependent phospholipid binding	STRING	Count	10	10	Membrane raft	DAVID	Count	7	9
		Enrich-ment	0.492 108	0.47069 2			Enrich-ment	213.45	301.97			p-value	3.80 E-02	1.30 E-02
		FDR q-value	3.2 E-15	2.21 E-08			FDR q-value	5.40 E-03	1.60 E-04			Benja-mini	2.90 E-01	1.20 E-01
Establishment of protein localization to membrane	STRING	Count	117	117	Extracellular matrix binding	STRING	Count	7	7	Actin cap	DAVID	Count	2	2
		Enrich-ment	0.256 069	0.23289 8			Enrich-ment	361.75	391.31			p-value	4.70 E-02	5.90 E-02
		FDR q-value	6.69 E-08	2.20 E-03			FDR q-value	2.60 E-04	2.00 E-04			Benja-mini	3.30 E-01	3.20 E-01
Cotranslational protein targeting to membrane	STRING	Count	79	79	Trans-membrane transporter activity	STRING	Count	110	109	Filamentous actin	DAVID	Count	3	3
		Enrich-ment	0.547 125	0.50323 3			Enrich-ment	4.45 E-01	7.89 E-01			p-value	5.3 E-02	7.90 E-02
		FDR q-value	1.08 E-15	6.69 E-09			FDR q-value	1.67 E-05	7.89 E-08			Benja-mini	3.50 E-01	3.80 E-01
Ion transmembrane transport	STRING	Count	117	116						Exocytic vesicle	STRING	Count	53	51
		Enrich-ment	9.21 E-01	6.93 E-01								Enrich-ment	0.9796 55	126.52
		FDR q-value	1.89 E-05	6.51 E-07								FDR q-value	2.92 E-05	8.60 E-04

Table S6. Impact of CHIP loss-of-function on the expression of proteins involved in ubiquitination. Related to Figure 3.

List of all proteins involved in protein degradation (via ubiquitination) for protein quality control identified in the SWATH-MS analyses of the CHIP cortical model and their fold changes and p-values.

**Protein**			**P/KO**		**WT/KO**		**P/WT**	
**Name**	**Symbol**	**Function**	**Fold change**	**p value**	**Fold change**	**p value**	**Fold change**	**p value**
Protein SGT1 homolog	SUGT1	Ubiquitination	0.979	3.79E-01	0.982	5.44E-01	0.998	9.50E-01
Ubiquitin-conjugating enzyme E2 D3	UBE2D3	E2	0.999	9.69E-01	0.881	2.20E-03	1.135	1.94E-03
Ubiquitin-conjugating enzyme E2 K	UBE2K	E2	0.996	8.69E-01	0.938	1.50E-02	1.062	6.18E-03
Ubiquitin carboxyl-terminal hydrolase 5	USP5	DUB	0.978	2.99E-01	0.941	4.30E-04	1.011	7.17E-01
Ubiquitin thioesterase OTUB1	OTUB1	DUB	1.014	3.44E-01	0.946	3.24E-02	1.074	1.13E-02
E3 ubiquitin-protein ligase HUWE1	HUWE1	E3	0.989	9.66E-01	0.961	4.84E-01	1.146	6.28E-01
Ubiquitin-conjugating enzyme E2 N	UBE2N	E2	1.081	5.40E-05	0.978	2.95E-01	1.106	2.19E-05
Polyubiquitin-C	UBC	Ubiquitin	1.062	2.78E-03	0.991	6.35E-01	1.073	1.30E-04
Ubiquitin carboxyl-terminal hydrolase 7	USP7	DUB	1.067	3.25E-01	0.994	9.11E-01	1.074	2.38E-01
Ubiquitin carboxyl-terminal hydrolase 14	USP14	DUB	1.066	9.51E-02	0.992	8.27E-01	1.075	9.12E-02
E3 ubiquitin-protein ligase UBR4	UBR4	E3	1.127	3.72E-01	1.001	9.91E-01	1.126	1.51E-01
Ubiquitin-like protein 4A	UBL4A	Ubiquitination co-factor	1.179	2.55E-01	1.036	8.10E-01	1.139	3.24E-01
Ubiquitin-conjugating enzyme E2 variant 2	UBE2V2	E2	1.041	3.48E-03	1.037	3.37E-02	1.005	6.76E-01
Ubiquitin-like modifier-activating enzyme 1	UBA1	E1	1.106	1.07E-02	1.037	1.54E-01	1.067	5.16E-02
Ubiquitin carboxyl-terminal hydrolase isozyme L1	UCHL1	E3 and DUB	1.047	4.42E-02	1.074	1.94E-03	0.976	3.95E-02
Ubiquitin carboxyl-terminal hydrolase	UCHL5	DUB	1.207	1.70E-01	1.176	1.48E-02	1.027	8.24E-01
Probable ubiquitin carboxyl-terminal hydrolase FAF-X	USP9X	DUB	0.439	2.55E-06	0.487	4.80E-06	0.902	6.52E-01
Ubiquitin-like modifier-activating enzyme 6	UBA6	E1	0.679	5.56E-03	0.625	2.04E-03	1.087	6.22E-01
Ubiquitin-like-conjugating enzyme ATG3	ATG3	E2	0.768	1.64E-01	0.764	1.10E-03	1.006	9.80E-01
Ubiquitin-conjugating enzyme E2 L3	UBE2L3	E2	0.951	2.49E-01	0.776	6.06E-05	1.227	3.30E-04
Ubiquitin-fold modifier 1	UFM1	Ubiquitin	0.976	8.45E-01	0.781	1.29E-01	1.251	2.97E-01
Small ubiquitin-related modifier 1	SUMO1	Ubiquitination			0.798	1.20E-04	1.013	8.01E-01
SMT3 suppressor of mif two 3 homolog 3	SUMO3	Ubiquitination	0.864	2.05E-08	0.743	1.27E-12	1.164	5.88E-08
Ubiquitin-like protein 5	UBL5	Ubiquitin	0.920	6.49E-01	0.815	2.95E-01	1.129	5.49E-01
Proteasomal ubiquitin receptor ADRM1	ADRM1	Regulates DUB	0.856	3.90E-01	0.842	1.36E-01	1.017	9.40E-01
Ubiquitin carboxyl-terminal hydrolase	UCHL3	DUB	0.991	7.68E-01	0.856	2.50E-04	1.159	4.27E-05
Ubiquitin carboxyl-terminal hydrolase 11	USP11	DUB	0.977	7.05E-01	0.870	6.11E-02	1.123	4.19E-02
E3 ubiquitin-protein ligase NEDD4-like	NEDD4L	E3	1.151	5.62E-02	0.872	8.66E-02	1.320	8.06E-05
Ubiquitin-conjugating enzyme E2 D3	UBE2D3	E2	0.999	9.69E-01	0.881	2.20E-03	1.135	1.94E-03

Table S7. Impact of CHIP loss-of-function on the expression of proteins involved in protein folding. Related to Figure 3.

List of all proteins involved in protein refolding (i.e. the chaperone system) for quality control identified in the SWATH-MS analyses of the CHIP cortical model and their fold changes and p-values.

**Protein**				**P/KO**		**WT/KO**		**P/WT**	
**Name**	**Symbol**	**HSP Family**	**Function**	**Fold change**	**P value**	**Fold change**	**P value**	**Fold change**	**P value**
Heat shock protein beta-1	HSPB1	HSP27	Chaperone	0.503	1.20E-07	0.456	1.76E-09	1.104	1.84E-01
60 kDa heat shock protein	HSPD1	HSP60	Chaperonin	1.074	6.60E-04	1.131	1.95E-03	0.951	1.09E-01
Heat shock 70 kDa protein 1B	HSPA1B	HSP70	Chaperone	1.096	5.66E-05	1.059	8.19E-03	1.036	9.12E-02
Heat shock 70 kDa protein 4	HSPA4	HSP70	Chaperone	1.018	7.52E-01	1.064	3.80E-04	1.020	1.25E-01
Heat shock 70 kDa protein 4L	HSPA4L	HSP70	Chaperone	1.018	7.52E-01	1.160	8.76E-03	0.930	3.08E-01
78 kDa glucose-regulated protein*	HSPA5	HSP70	Chaperone	0.885	6.59E-07	0.874	3.43E-06	1.014	4.33E-01
Heat shock cognate 71 kDa protein	HSPA8	HSP70	Chaperone	0.996	7.19E-01	1.076	3.44E-05	0.926	3.35E-05
Stress-70 protein	HSPA9	HSP70	Chaperone	1.135	6.28E-06	1.270	1.16E-11	0.894	1.72E-06
Heat shock 70 kDa protein 12A	HSPA12A	HSP70	Chaperone	1.692	5.90E-10	1.504	1.20E-02	1.126	2.91E-01
Heat shock 70 kDa protein 12B	HSPA12B	HSP70	Chaperone	0.866	4.77E-08	1.147	2.15E-03	0.755	4.01E-06
Heat shock 70 kDa protein 13	HSPA13	HSP70	Chaperone	0.956	1.81E-01	0.936	5.77E-02	1.021	4.69E-01
10 kDa heat shock protein	HSPE1	HSP70	Co-chaperonin	1.103	1.20E-08	1.145	4.17E-08	0.964	1.33E-02
Hsp70-binding protein 1	HSPBP1	HSP70	Co-chaperone	0.931	2.09E-02	0.912	1.49E-02	1.021	4.69E-01
Heat shock protein HSP 90-alpha	HSP90AA1	HSP90	Chaperone	1.084	1.70E-04	1.157	9.10E-06	0.938	9.27E-03
Endoplasmin	HSP90B1	HSP90	Chaperone	0.858	1.10E-10	0.925	2.86E-05	0.928	2.28E-06
Heat shock protein HSP 90-beta	HSP90AB	HSP90	Chaperone	1.021	6.25E-01	1.144	6.30E-03	0.894	1.37E-02
Heat shock protein HSP 90-beta	HSP90AB1	HSP90	Chaperone	1.021	6.25E-01	1.144	6.30E-03	0.894	1.37E-02
Heat shock protein 105 kDa	HSPH1	HSP105	Co-chapeone	1.092	4.35E-03	1.116	6.00E-04	0.980	1.81E-01
Large proline-rich protein BAG6	BAG6		Chaperone	1.279	1.09E-02	1.134	1.22E-01	1.129	2.70E-02
Tubulin-specific chaperone A	TBCA		Chaperone	1.017	3.50E-01	0.819	3.30E-07	1.243	3.15E-08
Hsp90 co-chaperone Cdc37	CDC37		Co-chaperone	1.037	5.02E-01	0.968	6.06E-01	1.071	1.54E-01
Cytochrome c oxidase copper chaperone	COX17		Chaperone	1.304	3.34E-02	1.302	7.81E-02	1.003	9.86E-01
DnaJ homolog subfamily A member 1	DNAJA1		Co-chaperone	0.965	4.63E-01	0.971	5.10E-01	0.994	9.01E-01

Table S8. GO terms associated to the proteomic changes detected in our CHIP KO cortical neuronal model and a dataset of injury-induced membrane protein changes. Related to Figure 3.

GO terms (molecular function) significantly associated to an injury-induced proteomic dataset previously published by Sønder et al., 2019 ("reference dataset") (A) and to our SWATH-MS analysis (P/KO, B, WT/KO, C, and P/WT, D) derived from GOrilla software. For the analyses, both a background list (all proteins identified) and a target list (proteins with ≤0.67 and ≥1.5 fold change). For each associated GO term a p-value, FDR q-value and Enrichment score is given. Refer to *Methods* for more detail. GO terms in bold were found in the Reference dataset, P/KO and WT/KO, but not in the P/WT analysis.

Analysis	GO term	Description	p-value	FDR q-value	Enrichment (N, B, n, b)
**Reference dataset**	GO:0003779	**Actin binding**	4.14E-11	7.12E-8	2.14 (1338,69,443,49)
	GO:0008092	Cytoskeletal protein binding	2.52E-7	1.08E-4	1.61 (1338,135,443,72)
	GO:0003743	Translation initiation factor activity	4.06E-8	3.49E-5	2.64 (1338,24,443,21)
	GO:0051015	Actin filament binding	5.31E-8	3.04E-5	2.20 (1338,44,443,32)
	GO:0017048	Rho GTPase binding	1.57E-6	5.39E-4	3.02 (1338,12,443,12)
	GO:0015291	Secondary active transmembrane transporter activity	1.48E-5	4.25E-3	2.52 (1338,18,443,15)
	GO:0008135	Translation factor activity, RNA binding	3.07E-5	7.54E-3	2.01 (1338,36,443,24)
	GO:0022804	Active transmembrane transporter activity	8.72E-5	1.87E-2	1.85 (1338,44,443,27)
	GO:0048365	Rac GTPase binding	1.38E-4	2.64E-2	3.02 (1338,8,443,8)
	GO:0004812	Aminoacyl-tRNA ligase activity	2.90E-4	4.99E-2	2.31 (1338,17,443,13)
	GO:0016875	Ligase activity, forming carbon-oxygen bonds	2.90E-4	4.54E-2	2.31 (1338,17,443,13)
	GO:0044325	Ion channel binding	3.35E-4	4.80E-2	2.16 (1338,21,443,15)
	GO:0140101	Catalytic activity, acting on a tRNA	3.39E-4	4.49E-2	2.10 (1338,23,443,16)
	GO:0015238	Drug transmembrane transporter activity	6.08E-4	7.46E-2	2.37 (1338,14,443,11)
	GO:0030165	PDZ domain binding	8.83E-4	1.01E-1	2.68 (1338,9,443,8)
**P/KO**	GO:0003779	**Actin binding**	8.63E-6	1.94E-2	2.41 (2049,100,221,26)
	GO:0005509	Calcium ion binding	3.24E-5	3.64E-2	2.46 (2049,83,221,22)
	GO:0005544	Calcium-dependent phospholipid binding	3.50E-5	2.62E-2	5.90 (2049,11,221,7)
	GO:0005200	Structural constituent of cytoskeleton	6.22E-5	3.49E-2	2.96 (2049,47,221,15)
	GO:0030280	Structural constituent of epidermis	1.32E-4	5.94E-2	9.27 (2049,4,221,4)
	GO:0008092	Cytoskeletal protein binding	2.41E-4	9.03E-2	1.67 (2049,239,221,43)
	GO:0098641	Cadherin binding involved in cell-cell adhesion	2.45E-4	7.86E-2	6.62 (2049,7,221,5)
**WT/KO**	GO:0003779	**Actin binding**	3.32E-4	1.85E-1	1.96 (2040,98,276,26)
	GO:0005200	Structural constituent of cytoskeleton	4.60E-5	1.03E-1	2.73 (2040,46,276,17)
	GO:0005544	Calcium-dependent phospholipid binding	1.57E-4	1.75E-1	4.70 (2040,11,276,7)
	GO:0048018	Receptor ligand activity	2.69E-4	2.00E-1	3.13 (2040,26,276,11)
	GO:0050840	Extracellular matrix binding	3.42E-4	1.53E-1	4.93 (2040,9,276,6)
	GO:0005102	Signaling receptor binding	7.15E-4	2.66E-1	1.60 (2040,194,276,42)
	GO:0098641	Cadherin binding involved in cell-cell adhesion	7.29E-4	2.32E-1	5.28 (2040,7,276,5)
	GO:0030545	Receptor regulator activity	8.24E-4	2.30E-1	2.80 (2040,29,276,11)
**P/WT**	GO:0030280	Structural constituent of epidermis	2.51E-5	5.61E-2	13.99 (2043,4,146,4)
	GO:0055102	Lipase inhibitor activity	7.40E-4	8.28E-1	8.00 (2043,7,146,4)
	GO:0005201	Extracellular matrix structural constituent	7.40E-4	5.52E-1	8.00 (2043,7,146,4)

Table S9. GO terms associated to the membrane proteomic changes in our CHIP cortical neuronal model. Related to Figure 4.

GO terms (biological process) significantly associated to the membrane proteomic changes in both P/KO and WT/KO analyses and excluded from P/WT. Membrane proteins identified by SWATH-MS were selected using IPA software. A background list of all membrane proteins and their fold changes was created for all SWATH-MS analyses (P/KO, WT/KO and P/WT), which consisted of 247, 244 and 245 proteins, respectively. Additionally, a target list of significantly changed membrane proteins (≤0.67 and ≥1.5 fold change) was also made for each dataset (P/KO, WT/KO and P/WT), along with fold changes, consisting of 47, 54 and 26 proteins, respectively. These lists were analysed by DAVID software to determine associated GO terms. Refer to Methods for more details.

GO term (biological process)	Analysis	Count	p-value	Benjamini
Neurotransmitter secretion	P/KO	4	3.20E-4	6.10E-2
	WT/KO	3	1.20E-3	1.60E-1
Cell adhesion	P/KO	7	1.20E-3	1.40E-1
	WT/KO	4	1.90E-2	5.50E-1
Cell-matrix adhesion	P/KO	4	1.70E-3	1.50E-1
	WT/KO	3	1.60E-2	5.40E-1
Positive regulation of vesicle fusion	P/KO	2	1.80E-2	4.00E-1
	WT/KO	2	1.60E-2	5.80E-1
Vesicle organization	P/KO	2	7.10E-2	6.70E-1
	WT/KO	2	6.30E-2	7.30E-1
Positive regulation of bone mineralization	P/KO	2	8.80E-2	7.40E-1
	WT/KO	2	9.60E-2	8.00E-1

Table S10. List of primers used for qRT-PCR. Related to STAR Methods.

Primers for qRT-PCR experiments were designed using the Roche UPL Assay design centre. When available these were intron-spanning. Their sequence is list, as well as probe required and the tested geometric efficiency.

Target gene	UPL Probe #	Forward sequence	Reverse sequence	Efficiency
hCDK6	85	gaactaggcaaagacctacttctga	ggtgggaatccaggttttct	2.161
hNanog	87	tctccaacatcctgaacctca	ttgctattcttcggccagtt	2.004
hOct4	78	tgccgtgaaactggagaag	gcttggcaaattgttcgagt	2.015
hVimentin	11	agatggcccttgacattgag	cagggaggaaaagtttggaa	1.844
hNestin	65	acctgtgccagcctttctta	gccaaggtaggggtacgg	2.154
hOtx2	86	aacctcccatgaggctgtaa	ggtggacaggttcagagtcc	2.007
hEmx1	25	ctctccgagacgcaggtg	ttcttctgctcggactcagg	2.015
hPax6	20	tcaccatggcaaataacctg	cagcatgcaggagtatgagg	1.980
hFoxG1	58	tactaccgcgagaacaagca	tcacgaagcacttgttgagg	2.075
hTbr2	69	gagtcggcaggtgggtag	tcttccgaggggaaggtaa	1.953
hTbr1	31	ttcaaataacaatgggcagatg	gtctcagggaaagtgaacgtct	2.156
hCtip2	82	ccgccagagatagggttttt	ctggtcatgcacaacctcag	2.032
hCux1	20	agaggccactgccctattct	ctggagatgatggaaagcagt	2.019
hBrn2	80	ctttgcaggcgagtaaccag	ttctagctatcacacactctcctca	1.967
hSNCA	68	gagggagtggtgcatggt	tgctgtcacacccgtcac	Devine et al., 2011
hStub1	70	gttcgtgggccgaaagta	ggcccggttggtgtaata	1.874
hTBP	87	gaacatcatggatcagaacaaca	atagggattccgggagtcat	2.005
